# Rhizosphere Engineering of Biocontrol Agents Enriches Soil Microbial Diversity and Effectively Controls Root-Knot Nematodes

**DOI:** 10.1007/s00248-024-02435-7

**Published:** 2024-09-28

**Authors:** K. Vinothini, S. Nakkeeran, N. Saranya, P. Jothi, J. Infant Richard, Kahkashan Perveen, Najat A. Bukhari, Bernard R. Glick, R. Z. Sayyed, Andrea Mastinu

**Affiliations:** 1 Department of Plant Pathology, Centre for Plant Protection Studies, Tamil Nadu Agricultural University, Coimbatore, 641 003 India; 2 Department of Plant Molecular Biology and Bioinformatics, Centre for Plant Molecular Biology and Biotechnology, Tamil Nadu Agricultural University, Coimbatore, 641003 India; 3 Department of Nematology, Centre for Plant Protection Studies, Tamil Nadu Agricultural University, Coimbatore, 641 003 India; 4 Department of Botany & Microbiology, College of Science, King Saud University, P.O. Box-22452, 11495 Riyadh, Saudi Arabia; 5 Department of Biology, University of Waterloo, Waterloo, ON N2L 3G1 Canada; 6 Department of Biological Sciences and Chemistry, College of Arts and Science, University of Nizwa, Nizwa, 616 Sultanate of Oman; 7 Department of Molecular and Translational Medicine, Division of Pharmacology, University of Brescia, Viale Europa 11, 25123 Brescia, Italy

**Keywords:** Biodiversity, Biocontrol agent, Amplicon sequencing, Nematode management, Soil microbiome, *Trichoderma koningiopsis*

## Abstract

The root-knot nematode (RKN) causes significant yield loss in tomatoes. Understanding the interaction of biocontrol agents (BCAs)-nematicides-soil microbiomes and RKNs is essential for enhancing the efficacy of biocontrol agents and nematicides to curb RKN damage to crops. The present study aimed to evaluate the in vitro effectiveness of BACa and nematicide against RKN and to apply the amplicon sequencing to assess the interaction of *Bacillus velezensis* (VB7) and *Trichoderma koningiopsis* (TK) against RKNs. Metagenomic analysis revealed the relative abundance of three phyla such as Proteobacteria (42.16%), Firmicutes (19.57%), and Actinobacteria (17.69%) in tomato rhizospheres. Those tomato rhizospheres treated with the combined application of *B. velezensis* VB7 + *T. koningiopsis* TK and RKN had a greater frequency of diversity and richness than the control. RKN-infested tomato rhizosphere drenched with bacterial and fungal antagonists had the maximum diversity index of bacterial communities. A strong correlation with a maximum number of interconnection edges in the phyla Proteobacteria, Firmicutes, and Actinobacteria was evident in soils treated with both *B. velezensis* VB7 and *T. koningiopsis* TK challenged against RKN in infected soil. The present study determined a much greater diversity of bacterial taxa observed in tomato rhizosphere soils treated with *B. velezensis* VB7 and *T. koningiopsis* TK than in untreated soil. It is suggested that the increased diversity and abundance of bacterial communities might be responsible for increased nematicidal properties in tomato plants. Hence, the combined applications of *B. velezensis* VB7 and *T. koningiopsis* TK can enhance the nematicidal action to curb RKN infecting tomatoes.

**Supplementary Information:**

The online version contains supplementary material available at 10.1007/s00248-024-02435-7.

## Introduction

Soil microorganisms are a vital component of soil and plant health. Naturally, soil contains a vast and diverse community of microorganisms, which governs both direct and indirect roles in the ecosystem to promote plant growth and suppress the proliferation of soil-borne plant pathogens through antagonistic activity [1]. For improving crop productivity, the soil microbiome promotes equilibrium and creates stable agrobiodiversity below ground [2]. Further, to understand the relationship and interaction between the soil microbiome and plants, metagenomic approaches have been used to analyze the diversity of the soil microbiome. These diverse soil microbiomes coexist with plant parasitic nematodes, microbial pathogens, and beneficial microbes. These beneficial microbes serve as a potential source of bio-control agents against plant parasitic nematodes [3]. Plant-parasitic RKNs are a significant concern for agriculture worldwide, infecting many crops [4, 5].

The annual loss due to the infestation of plant-parasitic nematodes is estimated to be ~ 14% of crop production [6], thus contributing ~ 30 to ~ 50% of crop loss globally [7, 8]. As a sedentary endoparasite, the life cycle of an RKN gets completed within plant tissue by developing giant cells in roots, which results in the formation of root galls and thus interferes with the plant’s metabolic processes [9, 10]. At present, managing plant parasitic nematodes in an eco-friendly manner remains challenging. Due to the significant economic impact of parasitic nematodes, various nematode-management techniques, including chemical nematicides, have been developed and used commercially to mitigate the infection of plants by nematodes [11].

Using bioagents compatible with the plant-beneficial microbes in the rhizosphere can positively impact the environment and become an effective, sustainable strategy for managing plant parasitic nematodes (PPN). Some bacterial biocontrol agents can hyperparasitize various stages of RKNs and thus reduce the hatching frequency of nematode eggs. In contrast, other biocontrol agents can induce the synthesis of plant-protective secondary metabolites, plant systemic resistance, and the production of phytohormones, antibiotics, and volatile organic compounds [12, 13]. The bacterial antagonists also reduce the invasion of PPN into plant roots [14], produce endospores, bind to nematode cuticles, induce the plant’s defense mechanism [15], and suppress the nematode infestation in tomatoes [16]. Further, among the fungal antagonists, *Trichoderma* species are widely considered exploitable fungal biocontrol agents to curb PPN [17]. Trichoderma’s mechanism of action includes the production of defensive metabolites, enzymes, and antioxidant compounds that potentially provide physical and chemical protection against RKN [18, 19]. RKN and soil biota interactions might be parasitic, symbiotic, communalistic, or hostile. However, understanding the diversity of bacterial microbiomes will aid in acquiring knowledge regarding the interaction between soil microbiomes and the homeostasis of soil ecosystems for preventing pest attacks and improving crop growth [20]. Further, nematicidal activity and the efficacy of applied biocontrol agents and soil-residing biota could be influenced by microbial interactions in the rhizosphere ecosystem with a wide range of activities, including antagonisms to syntrophic and mutualistic interactions [8].

The rhizospheric microbial diversity strengthens the resistance level of plant systems and disrupts the activity of soil-borne pathogens, including plant-parasitic nematodes [21]. Thus, it has been suggested that the best way to manage and prevent RKN infestation is to modify the composition of the rhizosphere microbial population and thereby retain the soil health [22]. Realizing the significance of microbial communities, their diversity in the rhizosphere was investigated using culture-dependent and -independent strategies. However, both techniques have certain limitations. To overcome these limitations, high-throughput sequencing (HTS) and shotgun metagenomic sequencing were carried out to facilitate the knowledge of the diversity of unculturable microbial communities to elaborate their functional network by monitoring the V1-V9 hypervariable region in bacteria [23]. Many researchers have also elaborated on the diversity of bacterial microbiomes [5, 24–27] residing in healthy and diseased tomato plants under field conditions. However, investigations of the interactions of rhizosphere microbial diversity and their role in curbing PPN are only in their infancy.

On the other hand, nematode microbiome studies revealed the diverse nature of various taxa involved in suppressing PPN [10, 28]. The knowledge of RKN-infected and healthy tomato roots explains the variations in the diversity of microbial communities associated with parasitism and antagonistic activity [29]. Although the prior research findings have demonstrated the role of microbes in inhibiting plant-parasitic nematodes, attempts made to identify the dominant group of soil bacteria associated with nematode prevalence in the field remain to be explored. This situation has encouraged us to investigate the current knowledge on the linkages between bacterial rhizobiome related to plant parasitic nematodes, biocontrol agents, and nematicides used to manage nematodes. The underlying idea behind this study has been that the individual or combined applications of antinemic biocontrol agents with chemical nematicides to suppress RKN might affect bacterial rhizobiome diversity or promote changes in root biology with effects on specialized rhizosphere organisms. Before this study, there was no evidence regarding the diversity analysis of microbial communities following the combined application of two biocontrol agents to tomato plants at the field level to manage RKN infestation. The present study aimed to differentiate the abundance and diversity of various bacterial communities distributed in biocontrol agent-treated rhizosphere soil and RKN-infected rhizosphere soil at various taxonomical levels thorough amplicon sequencing. Therefore, we investigated the diversity of the bacterial microbiome in tomato rhizosphere soil through the combined application of *B. velezensis* VB7 and *T. koningiopsis* TK against RKN infestation to assess the changes in bacterial abundance and diversity in treated and untreated rhizosphere soils using the next generation sequencing (NGS)-based amplicon sequencing by measuring the abundance of the operational taxonomic units (OTUs) in the rhizosphere.

## Materials and Methods

### Preparation of Nematode Inoculum

Eggs collected from severely infected galled tomato roots were used as the nematode inoculum. The eggs from the gelatinous matrix were separated by chopping roots into 1–2 cm pieces. Chopped roots were placed in a 500-ml plastic container containing 1.5% chlorine solution and shaken forcefully for 3 min. The suspension was then rinsed six times with running tap water through a 250-µm aperture mesh sieve, with the eggs being collected on a sieve with a 20-µm aperture. After 4 days of incubation at 28 ± 2 °C, the hatched second-stage juveniles (J2) were collected from the egg suspension using a modified Baermann dish and utilized for subsequent experiments.

### Testing the Nematicidal Activity of Bacterial and Fungal Antagonists Against RKN

The bacterial antagonist *B. velezensis* VB7 (MW301630) and the fungal antagonist *T. koningiopsis* TK (KX 555650) were obtained from the Department of Plant Pathology, Tamil Nadu Agricultural University (TNAU), Coimbatore, India. The effect of biocontrol strains such as *B. velezensis* VB7 and *T. koningiopsis* TK with nematicide activity on the second-stage juveniles (J2) of *M. incognita* was evaluated by the nematode mortality rate and the hatching ability of egg mass. The bacterial culture (*B. velezensis* VB7) was inoculated into LB broth and maintained in an orbital shaker at 150 rpm, at room temperature (28 ± 2 °C), for 48 h to ensure uniform bacterial growth. The fungal discs (*T. koningiopsis*) were inoculated into Potato Dextrose Broth (PDB) at 28 °C and maintained for 5 days. Supernatants were collected by centrifugation of culture suspensions at 10,000 rpm for 10 min at 5 °C, which contained no viable cells. The supernatants were collected, and 3 ml of cell-free culture filtrates of biocontrol agent was poured separately into a 6-cm (diameter) Petri dish. 2-egg masses of *M. incognita* were added using a camel hair brush for each replication and incubated at 28 ± 2 °C. The hatching percentage was observed at 24 h, 48 h, 72 h, and 96 h after incubation. Sterile water was used as a control. Similarly, for the mortality assay, hatched second-stage juveniles (J2) were adjusted to the concentration of 50 juveniles ml^−1^. Two milliliters of nematode suspension (100 juveniles) was inoculated into 6-cm Petri plates containing 3-ml cell-free culture filtrates of the biocontrol agents. Three replications were maintained and incubated at room temperature (28 ± 2 °C). After 24 h, 48 h, 72 h, and 96 h, the number of surviving and dead individuals was recorded using a 1-ml Hawksley counting slide. The formula calculated the percentage mortality:

$$\text{Mortality }\left({\%}\right)=\frac{{C}_{1}-{C}_{2}}{{C}_{1}} \times 100\dots$$

where *C*_1_ is the number of live juveniles released, and *C*_2_ is the number of live juveniles counted. Analysis of variance was used to conduct a statistical analysis of the data collected from the in vitro experiments. For analysis of variance (ANOVA), Tukey’s test was performed using SPSS 20.0 (IBM, SPSS statistics 20) with a significance threshold of 0.05.

### Collection of Samples

Rhizosphere soils were collected by a random sampling technique at 35 days after combined applications of 1% *Trichoderma koningiopsis* TK formulation (3 × 10^8^ cfu/g) [30] and a liquid formulation of *Bacillus velezensis* VB7 (5 × 10^8^ cfu/ml) as per the protocol of Vinodkumar et al. [31] from six different treatments including T_1 _- *B. velezensis* VB7 alone; T_2_ - *B. velezensis* VB7 + RKN; T_3_ - *T. koningiopsis* alone; T_4_ - *T. koningiopsis* + RKN; T_5_ - *B. velezensis* VB7 + *T. koningiopsis* TK; T_6_ - untreated control in RKN-infected tomato fields at Thondamuthur in Coimbatore province, Tamil Nadu, India (GPS coordinates: 10.5484° N 76.2857° E), by maintaining three biological replicates for each sample. Each replicate was laid over an area of 40 m^2^. Collected samples were stored in sterile polypropylene bags, immediately placed on an ice box, transported to the laboratory, and stored at − 80 °C until processing.

### PCR Amplification of 16S rRNA and Purification

Metagenomic DNA was separately extracted from the collected rhizosphere soils for all treatments, including T_1_ - *B. velezensis* VB7 alone; T_2_ - *B. velezensis* VB7 + RKN; T_3_ - *T. koningiopsis* alone; T_4_ - *T. koningiopsis* + RKN; T_5_ - *B. velezensis* VB7 + *T. koningiopsis* TK; T_6_ - untreated control in RKN-infected tomato fields, using Power Soil DNA Isolation Kit (QIAGEN, India) 30 days after planting. Quantitative and qualitative analysis of DNA was performed using the nanodrop method, followed by 1% agarose gel electrophoresis using a TAE electrode buffer. A total of 50 ng DNA from each sample was subjected to 16S rRNA PCR gene amplification using V1-V9 region-specific primers of 27F 5′AGAGTTTGATCMTGGCTCAG3′ and 1492R 5′TACGGYTACCTTGTTACGACTT3′. The amplicons obtained from the samples were confirmed by agarose (1%) with EtBr gel electrophoresis. The PCR products were purified using 1.6X Ampure XP beads (Beckmann Coulter, USA).

#### Library Preparation and Sequencing of DNA Product

A total of ~ 50 ng from each amplicon DNA was end-repaired (NEBnext ultra II end repair kit; New England Biolabs, MA, USA) and cleaned with 1 × AmPure beads. Barcoding adapter ligation (BCA) was performed with NEB blunt/TA ligase and cleaned with 1 × AmPure beads. Qubit quantified adapter-ligated DNA samples were barcoded using PCR reactions, pooled at equimolar concentration, and end-repair was performed using NEBnext ultra II end repair kit (New England Biolabs, MA, USA) and cleaned. Adapter ligation (AMX) was performed for 15 min using NEB blunt/TA ligase (New England Biolabs, MA, USA). Library mix was cleaned using Ampure beads and then eluted in 15 μl of elution buffer (GENE, Bengaluru, India). Sequencing was performed for prokaryotic and eukaryotic organisms through the Oxford Nanopore sequencing method using MinION flow cell R9.4 (FLO-MIN106). Nanopore raw reads (“fast5” format) were base-called (“fastq5” format) and de-multiplexed using Guppy1 v2.3.4.

#### Processing of Sequencing Data and Taxonomic Profiling

Sequencing data were processed with the Guppy v2.3.4 tool kit for base calling the sequencing data to generate a pass read. The adapter and barcode sequences were trimmed using the Porechop tool. The reads were filtered by size using SeqKit software ver. 0.10.0, and the average Phred quality score was assessed using the SILVA database. A comprehensive taxonomic microbial community analysis was performed on each set’s processed reads. The obtained rRNA reads were imported into Qiime2 in the Single End Fastq Manifest Phred33 input format for diversity analysis. The readings were de-duplicated and then grouped into operational taxonomic units (OTUs) based on their 97% similarity [32]. BLAST with QIIME (categorize-consensus-search) was used to classify representative sequences using percent identity 0.97 against the SILVA full-length 16S database. The long-read amplicon data was sequenced using Nanopore MinION and validated against SILVA databases to determine their proper classification. The relative microbial abundances were determined to categorize the microbial community of each sample according to their taxonomic profile (Kingdom, Phylum, Class, Order, Family, Genus, Species) in a stacked bar chart. Singletons and sequences classified as mitochondria, chloroplast, Archaea, and unassigned sequences were removed. Only the top 20 bacterial OTUs were used for the analysis.

### Diversity Index Analysis

The microbial diversity analysis was estimated by calculating the alpha and beta diversity indexes using the obtained OTU cluster. The alpha diversity was carried out using the evenness vector, Jaccard observed features vector [33], and Shannon vector [34] algorithms to determine the diversity and richness of the microbial community within a sample. In contrast, beta diversity represents the diversity between different samples using the Bray–Curtis dissimilarity statistic [10, 35]. Samples were compared with a comprehensive set of multivariate statistical tools, including PERMANOVA and visualization tools. According to Zheng and collaborators, OTU comparisons were performed using the Venn diagram package [25]. All of these indices in our samples were calculated using QIIME (version 1.7.0, http://qiime.org/1.7.0/). Principal coordinate (PCoA) plots were generated to determine the community structure using QIIME1 version 1.9.1 [36]. Statistical analyses were conducted using R statistical software with diverse sub-programs [37]. All significance tests were two-sided; *P* values < 0.05 were considered statistically significant. Gephi 0.10.1 was used to construct the co-occurrences co-efficient network using each sample’s mean average of OTUs.

#### The Taxonomic Abundance of the Microbial Population Through Cluster Heatmap

According to the abundance of information on microbial communities at the taxonomic level, the heatmap was constructed by clustering similar communities of each sample to determine the frequency of microbial communities.

#### Venn Diagrams

Venn diagrams were constructed to determine the relationship between bacterial communities in treated and untreated soil. They were compared at the individual as well as combined applications of bioagents and nematicide at the genus and species level by using the Muthor program and then submitted to VENNY (http://bioinfogp.cnb.csic.es/tools/venny/index.html) to show the shared and unique OTUs [29].

## Results

### Efficacy of Culture Filtrate of *B. velezensis* VB7 and *T. koningiopsis* TK Against Egg Hatching and Juveniles’ Mortality of Root-Knot Nematode *M. incognita* Under In Vitro Conditions

**Fig. 1:**
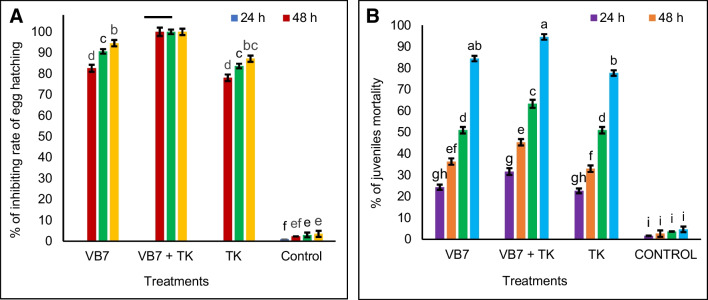
Efficacy of culture filtrate of *B. velezensis* and *T. koningiopsis* TK against egg hatching and juveniles’ mortality of root-knot nematode (RKN) *M. incognita* at different intervals, under in vitro conditions. **A** Percentage of juvenile mortality. **B** Percentage of inhibition of egg hatching. Means indicated by the same lower-case letters show significant and insignificant differences according to Tukey’s test at *P* ≤ 0.05. Each value is the average of three replicates, and the error bar indicates ± standard deviation (SD) (VB7 + RKN = *B. velezensis* VB7 + root-knot nematode, VB7 = *B. velezensis* VB7 alone, TK + RKN = *T. koningiopsis* TK + root-knot nematode, TK = *T. koningiopsis* TK alone, VB7 + TK + RKN = *B. velezensis* VB7 + *T. koningiopsis* TK + root-knot nematode; RKN, root-knot nematode (*M. incognita*))

The cultural filtrates of *B. velezensis* VB7 and *T. koningiopsis* TK and water (control, T_6_) were screened for their efficacy on egg hatching and juvenile mortality of *M. incognita* under in vitro conditions. *B. velezensis* VB7 combined with *T. koningiopsis* TK effectively inhibited 94.50% of juveniles’ mortality of *M. incognita* after 96 h exposure. *B. velezensis* VB7 alone accounted for 85.70% mortality, and the cultural filtrates of *T. koningiopsis* TK alone inhibited 77.45% mortality, whereas lower juveniles’ mortality of 4.6% was observed in control (T_6_ – Sterile water), respectively (Fig. 1A). The egg hatching rate of *M. incognita*, treated with *B. velezensis* VB7 + *T. koningiopsis* TK, was 0% of egg hatching with 100% reduction in egg hatching after a 96 h exposure, a noticeable drop (*P* < 0.05) compared with the control (T_6_). At the same time, the cultural filtrates of *B. velezensis* VB7 (T_1_) and *T. koningiopsis* TK (T_3_) alone showed 94.45% inhibition of egg hatching and 87.15%, respectively (Fig. 1B). The efficacy of the nematicide activity was correlated with the exposure time.

### OTU Identification and Taxonomic Annotation for Bacteria

Approximately 1,210,401 raw reads were generated from Illumina MiSeq sequencing of the six samples, with an average of 201,734 reads per sample. After quality control, 1,025,357 clean reads were obtained from the raw reads for six samples, with an average of 170,893 reads per sample. The quality-filtered reads were further size-filtered to obtain the classified OTU to retain 1200–1950 bp sequences for the V1-V9 region. In total, 16,892 sizes of filtered reads ranging from 865 to 3517 were identified after processing of QC filtered reads. A total of 3788 OTUs were identified as classified reads in all six samples. Among these, 746 OTUs (19.70%) were found in T1, 561 OTUs (14.80%) were present in T2, 291 OTUs (7.70%) were analyzed in T3, 737 OTUs (19.45%) were recorded in T4, 737 OTUs (19.45%) were observed in T5, and 716 OTUs (18.90%) were identified in T6, which were used for further analysis.

### Analysis of the Bacterial Community’s Composition

#### Relative Abundance

**Fig. 2:**
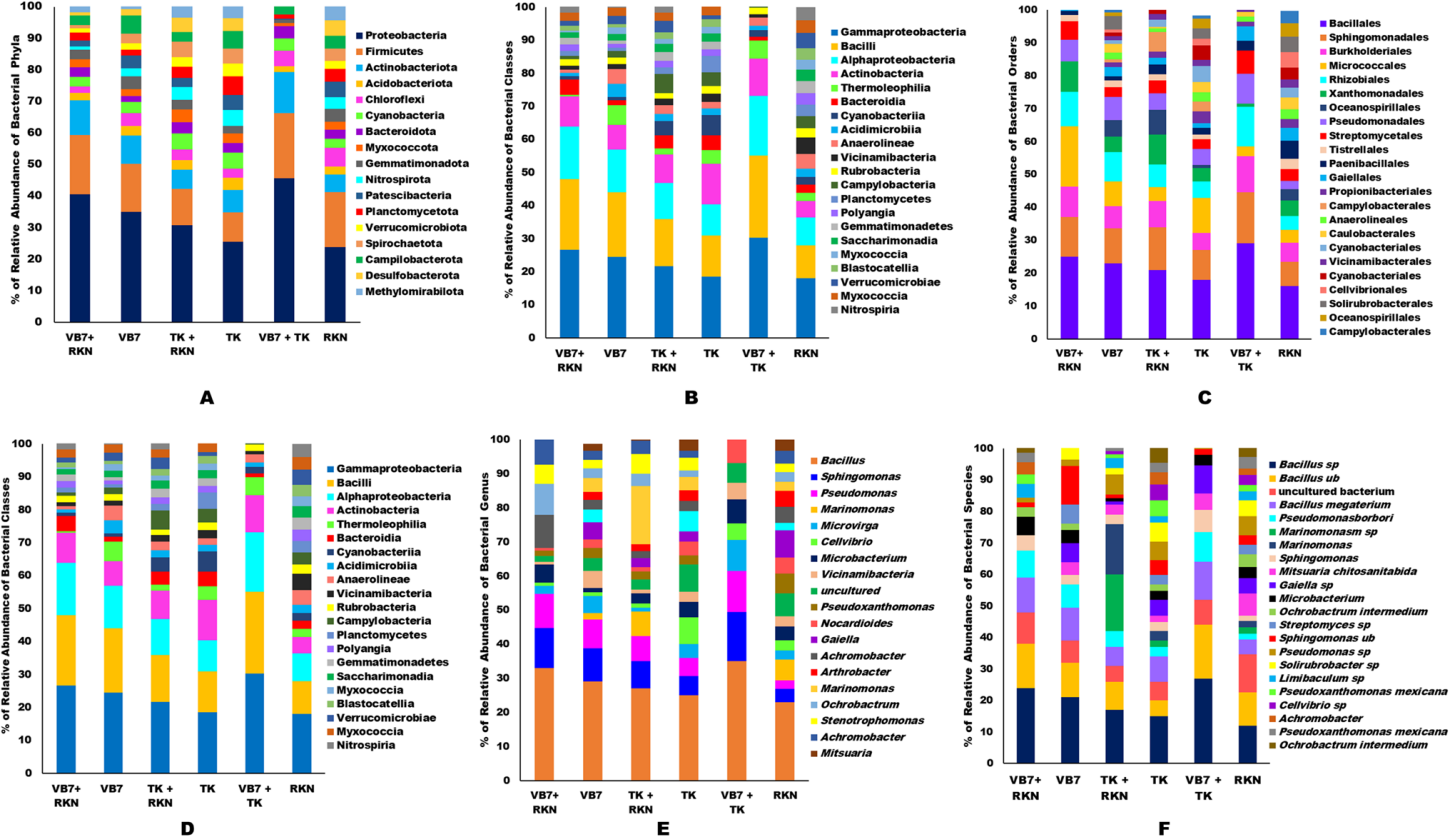
Relative abundances of rhizosphere bacteria communities distributed in treated and untreated rhizospheric soil. **A** Relative abundances of different bacterial phyla distributed in different treatments. **B** Relative abundances of different bacterial classes distributed in different treatments. **C** Relative abundances of various bacterial orders distributed in different treatments. **D** Relative abundances of various bacterial families distributed at different treatments. **E** Relative abundances of various bacterial genus distributed in different treatments. **F** Relative abundances of various bacterial species distributed in different treatments (VB7 + RKN = *B. velezensis* VB7 + root-knot nematode, VB7 = *B. velezensis* VB7 alone, TK + RKN = *T. koningiopsis* TK + root-knot nematode, TK = *T. koningiopsis* TK alone, VB7 + TK + RKN = *B. velezensis* VB7 + *T. koningiopsis* TK + root-knot nematode; RKN, root-knot nematode (*M. incognita*))

According to the microbial classification, 21 phyla, 48 classes, 105 orders, 135 families, 205 genera, and 252 species with different bacterial communities were identified from the tomato rhizosphere soil, irrespective of other treatments. Taxonomic annotation of each sample was grouped at each level to determine the proportion of relative abundance of each sample at different taxonomic classification levels. The abundance of each taxa level for each sample was represented in the stacked bar chart (Fig. 2A–E).

Comparative analysis of all the rhizosphere soil samples revealed the presence of major bacterial Phyla such as Proteobacteria, Firmicutes, Actinobacteriota, Acidobacteriota, Chloroflexi, Cyanobacteria, Bacteroidota, Myxococcota, Gemmatimonadota, Nitrospirota, Patescibacteria, Planctomycetota, Verrucomicrobiota, Spirochaetota, Campilobacterota, Desulfobacterota, and Methylomirabilota. Among the different phyla, Proteobacteria (42.16%), Firmicutes (19.57%), and Actinobacteria (17.69%) were the three predominant phyla in all six samples, including the RKN control (Fig. S1). The relative abundance of Proteobacteria was always much higher than Firmicutes and Actinobacteria; for example, Proteobacteria abundance was 52.59% in the tomato rhizosphere soils drenched with *B. velezensis* VB7 + *Trichoderma koningiopsis* TK. It increased to 40.71% in the tomato rhizosphere drenched with *B. velezensis* VB7 + RKN soil and 30.71% in *B. velezensis* VB7 alone inoculated soil. Similarly, the relative abundance of Proteobacteria was 30.67% in *T. koningiopsis* TK + RKN sample, while 25.03% of abundance was found in *T. koningiopsis* TK alone. Interestingly, the relative abundance of Proteobacteria in the rhizosphere soil of RKN-infected samples was 23.26%. The relative abundance of a second dominant phylum, Firmicutes, increased to 20.64% in tomato rhizosphere soils drenched with *B. velezensis* VB7 + *T. koningiopsis* TK. At the same time, *B. velezensis* VB7 + RKN soil had an abundance of 18.72% compared to 15.17% in *B. velezensis* VB7 alone drenched soil. However, RKN-infected soils had a lower abundance of Firmicutes, i.e., 15.38%. Further, the abundance of Firmicutes was comparatively higher in both *T. koningiopsis* TK + RKN (11.61%) and *T. koningiopsis* TK alone drenched soils (9.72%), compared to the RKN alone infested soils. Thus, the application of *B. velezensis* VB7 and *T. koningiopsis* TK significantly promoted the proliferation of Proteobacteria and Firmicutes (Fig. 2A). The relative abundance of Actinobacteria ranged from 5.80 to 13.00%, irrespective of all the treatments. Interestingly, the relative abundance of Actinobacteria was more abundant in rhizosphere soils drenched with *B. velezensis* VB7 + *T. koningiopsis* TK soil (13.69%). In contrast, *B. velezensis* VB7 + RKN soil treated soil had 11.00%, while soil drenched with *B. velezensis* VB7 alone had 9.47%, compared to the RKN-infested rhizosphere soil samples (2.61%).

Analysis of top 20 bacterial classes present in different rhizosphere soil samples revealed the existence of γ proteobacteria, Bacilli, α proteobacteria, Actinobacteria, Thermoleophilia, Bacteroidia, Cyanobacteria, Acidimicrobia, Anaerolineae, Vicinamibacteria, Rubrobacteria, Campylobacteria, Planctomycetes, Gemmatimonadetes, Saccharimonadia, Myxococcia, Blastocatellia, Verrucomicrobiae, Myxococcia, and Nitrospiria (Fig. S2 ). At the class level, γ proteobacteria (24.80%), α proteobacteria (21.61%), Bacilli (19.86%), and Actinobacteria (12.14%) were dominant in both treated and untreated rhizosphere samples. Examination of the relative abundance of γ proteobacteria from various rhizosphere soils indicated that *B. velezensis* VB7 combined with *T. koningiopsis* TK-treated soil had 30.48% of γ proteobacteria population. However, in *B. velezensis* VB7 + RKN soil, 26.62% γ proteobacteria abundance was found, whereas 24.48% was observed in *B. velezensis* VB7 alone treated soil. Similarly, *T. koningiopsis* TK-drenched soil had 18.5%. However, the population of γ proteobacteria was increased to 21.58% *T. koningiopsis* TK + RKN treatments. The soil drenched with *B. velezensis* VB7 + *T. koningiopsis* TK + RKN induced the proliferation of γ-Proteobacteria rather than the absence of RKN infection. However, 17.98% of γ proteobacteria abundance was found in RKN-infested soil, which was lower than in treated soil. Similarly, the relative abundance of α-proteobacteria in the rhizosphere soil treated with *B. velezensis* VB7 in the presence of RKN was 15.96%, compared to 19.06% in soil treated with *B. velezensis* VB7 alone. Moreover, the abundance increased to 24.97% in *B. velezensis* VB7 + *T. koningiopsis* TK + RKN soil. However, the *T. koningiopsis* TK + RKN treatment had 14.91%, whereas *T. koningiopsis* TK without RKN soil had 11.45% of the α-proteobacteria population. Thus, combining *B. velezensis* VB7 + *T. koningiopsis* TK + RKN treatment increased the α proteobacteria population density due to synergistic interaction between antagonistic biocontrol agents and the rhizosphere bacteriome. On the contrary, the relative abundance of α proteobacteria was comparatively lower in RKN infestation soil (8.45%) than in biocontrol agents treated soil. The maximum relative abundance of Bacilli was present in *B. velezensis* VB7 + *T. koningiopsis* TK + RKN treatment with the range of 24.35%. The lower population of was found in *B. velezensis* VB7 alone (19.5%) as compared to 21.52% in *B. velezensis* VB7 with RKN-infested soil. However, with the plant’s defense, the abundance of *Bacilli* was 14.50% in *T. koningiopsis* TK + RKN treated soil and 12.45% in *T. koningiopsis* TK alone drenched soil (Fig. 2B). The abundance of Bacilli was comparatively very low in RKN-infested soil (10.14%). The abundant population of Cyanobacteria was found in *T. koningiopsis* TK drenched rhizosphere soil (6.06%), followed by 4.65% in *T. koningiopsis* TK + RKN soil, whereas low in RKN soil 2.2%. The *B. velezensis* VB7 + *T. koningiopsis* TK showed a significant increase in the populations of γ proteobacteria and bacilli. However, the soil drenching of *B. velezensis* VB7 and *T. koningiopsis* TK with or without RKN association positively influenced the abundance of the Bacilli population. Similarly, the combined application of *B. velezensis* VB7 + *T. koningiopsis* TK with RKN and *B. velezensis* VB7 + RKN treatment positively impacted the abundance of α proteobacteria population. However, applying *T. koningiopsis* TK increased the presence of a Cyanobacteria population.

The bacterial orders include Bacillales, Sphingomonadales, Burkholderiales, Micrococcales, Rhizobiales, Xanthomonadales, Oceanospirillales, Pseudomonadales, Streptomycetales, Tistrellales, Paenibacillales, Gaiellales, Propionibacteriales, Campylobacterales, Anaerolineales, Caulobacterales, Cyanobacteriales, Vicinamibacterales, Cyanobacteriales, Cellvibrionales, Solirubrobacterales, and Campylobacterales were ranked higher abundance in the rhizosphere region of all different soil samples (Fig. S3). Among the top 20 orders, Bacillales (17.47%), Sphingomonadales (7.60%), Burkholderiales (7.60%), Micrococcales (6.42%), and Rhizobiales (5.17%) were the most prominent ones in rhizosphere soil abundance. The soil treated with *B. velezensis* VB7 + *T. koningiopsis* TK in RKN infestation had the most significant influence on the abundance of the Bacillales population, accounting for 29.33%, followed by 25.93% in *B. velezensis* VB7 + RKN soil, and 23.34% in *B. velezensis* VB7 only applied soil. On the other hand, *T. koningiopsis* TK + RKN had a relative abundance of 21.34% of Bacillales compared to 18.28% in *T. koningiopsis* TK alone drenched soil. However, a lower population of Bacillales was observed in RKN-infested soil at 16.43%, which was lower than the biocontrol agents’ drenched soil. A comparison of the relative abundance of Sphingomonadales order between different treatments indicated an abundance value of 15.43% in the soils applied with *B. velezensis* VB7 + *T. koningiopsis* TK in RKN soil. However, 9.59% of Sphingomonadales populations were found in *B. velezensis* VB7 + RKN soil and 6.64% in *B. velezensis* VB7 applied to the soil. Moreover, it was only 7.66% in RKN-infested soils. On the other hand, *T. koningiopsis* TK alone and *T. koningiopsis* TK + RKN treatment had abundances of 13.71% and 7.14%, respectively. A lower abundance of 5.82% was observed in RKN-infected soil alone. Similarly, a significant percentage of the rhizosphere population was found in *B. velezensis* VB7 and *T. koningiopsis* TK + RKN treatment, accounting for 12.46%, 8.49% in *B. velezensis* VB7 with RKN soil, and 4.51% in RKN infested soils, respectively. The maximum population of Rhizobiales of 10.54% was found in *B. velezensis* VB7 + RKN. However, the population was reduced in soil drenched with *T. koningiopsis* TK alone (5.20%) compared to *T. koningiopsis* TK + RKN (7.43%). Combined application of *B. velezensis* VB7 + *T. koningiopsis* TK might have contributed towards the proliferation of Rhizobiales populations in rhizosphere soil rather than a single application of the biocontrol agents (Fig. 2C).

Distribution of top 20 bacterial families in different soil samples includes Bacillaceae, Sphingomonadaceae, Xanthomonadaceae, Comamonadaceae, Pseudomonadaceae, Marinomonadaceae, Rhizobiaceae, Micrococcaceae, Microbacteriaceae, Geminicoccaceae Alcaligenaceae, Rhodobacteraceae, Beijerinckiaceae, Nocardioidaceae, Vicinamibacteraceae, Caulobacteraceae, Arcobacteraceae, Streptomycetaceae, Gaiellaceae, Gemmatimonadaceae, and Rhodanobacteraceae (Fig. S4). Among those families, Bacillaceae (17.08%), Sphingomonadaceae (8.25%), Xanthomonadaceae (4.10%), and Rhizobiaceae (4.30%) populations were dominant in the rhizosphere region. The *B. velezensis* VB7 + RKN soil constituted 33.69% of the Bacillaceae population compared to the 31.05% in *B. velezensis* VB7 treatment alone. Similarly, the soil drenched with *T. koningiopsis* TK in RKN constituted 27.49% of Bacillaceae, while 25.32% in *T. koningiopsis* TK alone applied to the soil. Moreover, a significant proportion of Bacillaceae was found with a *B. velezensis* VB7 + *T. koningiopsis* TK + RKN treatment. A lower population was noticed in RKN-infected tomato rhizosphere (21.45%). On the contrary, the relative abundance of Sphingomonadaceae was detected in *B. velezensis* VB7 + RKN with the range of 19.67% compared with 17.93% in *B. velezensis* VB7 alone drenched soil. In addition, the tomato rhizosphere soil drenched with *B. velezensis* VB7 + *T. koningiopsis* TK + RKN had a maximum relative abundance of Sphingomonodaceae of up to 23.93%. Though the abundance level was lower than the soils drenched with *T. koningiopsis* TK (i.e., 11.34%), it was higher than *T. koningiopsis* TK + RKN treatment (15.43%). However, the abundance of the Sphingomonodaceae population was low (9.87%) in the RKN-infected soil. Hence, the RKN-infected tomato rhizosphere soil drenched with *B. velezensis* VB7 + *T. koningiopsis* TK was comparatively higher in the population than when applying individual biocontrol agents. Similarly, the relative abundance of Rhizobiaceae was 7.70% in the *B. velezensis* VB7 + RKN compared to 6.05% in *B. velezensis* VB7 alone. However, in RKN soils, it was only 3.07%. But, the population of Rhizobiaceae in *T. koningiopsis* TK alone was 4.34% and 5.98% in *T. koningiopsis* TK + RKN soil. The highest abundance of the Rhizobiaceae population, i.e., 9.45%, was found in *T. koningiopsis TK* + *B. velezensis VB7* + *RKN* compared to 3.90% in untreated control soil (RKN). However, the Rhizobiaceae abundance is also high in the soils amended with bacterial and fungal antagonists in RKN soil. The relative abundance of the Pseudomonaceae was 12.14% and 10.89% in *B. velezensis* VB7 + RKN soils and *B. velezensis* VB7 TK alone treated soil, respectively. However, the abundance of Pseudomonaceae was 14.81% in *B. velezensis* VB7 + *T. koningiopsis* TK applied soils, as against 3.6% in RKN soils (Fig. 2D). Thus, in the presence of RKN, the population dynamics of different bacterial families were increased with the single or combined application of *B. velezensis* VB7 and *T. koningiopsis* TK.

The top 20 bacterial genera prevalent in rhizosphere soil were identified as *Bacillus*, *Sphingomonas*, *Pseudomonas*, *Marinomonas*, *Microvirga*, *Cellvibrio*, *Microbacterium*, *Vicinamibacteraceae*, *Pseudoxanthomonas*, *Nocardioides*, *Gaiella*, *Achromobacter*, *Arthrobacter*, *Marinomonas*, *Ochrobactrum*, *Stenotrophomonas*, *Achromobacter*, *and Mitsuaria* (Fig. S5). Three bacterial genera, including *Bacillus* (16.07%), *Sphingomonas* (5.56%), and *Pseudomonas* (3.6%), were observed in the highest numbers in all soil samples. The soil with *B. velezensis* VB7 + RKN contained 33.24% *Bacillus* genera compared to 29.24% in soils with *B. velezensis* VB7 alone. In addition, a *Bacillus* abundance of 39.70% was observed in soil drenched with *B. velezensis* VB7 + *T. koningiopsis* TK + RKN. *T. koningiopsis* TK + RKN treated soil had 27.24% *Bacillus* abundance against 25.25% in *T. koningiopsis* alone. At the same time, RKN-infected soil had a lower proportion of the *Bacillus* population, 35.87%. The abundance of *Sphingomonas* was lesser in *T. koningiopsis* TK + RKN and *T. koningiopsis* TK soil, i.e., 8.75% and 5.34% compared to *B. velezensis* VB7 + RKN and *B. velezensis* VB7 alone which was 11.75% and 9.45%, respectively. The maximum proportion of *Sphingomonas* was highly increased in *B. velezensis* VB7 + *T. koningiopsis* TK + RKN sample. But, the RKN-infected soil had a comparatively lower abundance of *Sphingomonas* (3.85%). The abundance of *Pseudomonas* in *B. velezensis* VB7 + RKN soil was 10.04% compared to 8.13% in *B. velezensis* VB7 alone. The abundance of *Pseudomonas* was 12.45% in *T. koningiopsis* TK + *B. velezensis* VB7 applied soils with the presence of RKN. At the same time, the *T. koningiopsis* TK + RKN soil had 8.75% *Pseudomonas* compared to 5.52% in *T. koningiopsis* TK alone and 2.54% in RKN-infected soil. The *Microvirga* genus had a comparatively higher percentage of B. velezensis VB7 + *T. koningiopsis* TK + RKN treatment, which was 9.24%. However, the abundance was decreased in *T. koningiopsis* TK + RKN soil to 1.20%. However, it was increased in *B. velezensis* VB7 alone applied to soil to 5.45%, followed by RKN-infected soil, which was 1.20%. The abundance was lower in both *B. velezensis* VB7 + RKN and *T. koningiopsis* TK + RKN soil (Fig. 2E).

The species diversity observed in various rhizosphere soils comprised of *Bacillus* sp., *B. megaterium*, *Pseudomonas borbori*, *Marinomonasm* sp., *Sphingomonas* sp., *Mitsuaria chitosanitabida*, *Gaiella* sp., *Microbacterium* sp., *Ochrobactrum intermedium*, *Streptomyces* sp., *Sphingomonas* sp., *Pseudomonas* sp., *Solirubrobacter* sp., *Limibaculum* sp., *Pseudoxanthomonas mexicana*, *Cellvibrio* sp., *Achromobacter* sp., and *Pseudoxanthomonas mexicana* (Fig. S6). *Sphingomonas* sp. and *Gaiella* sp. are considered uncultured bacterial species. The culturable *Bacillus* spp. was abundant in *B. velezensis* VB7 + *T. koningiopsis* TK + RKN soil (27.56% abundance), followed by 24.67% abundance of *B. velezensis* VB7 + RKN and 21.06% abundance in *B. velezensis* VB7. However, *T. koningiopsis* TK, with the presence of RKN, constituted the *Bacillus* spp., with an abundance of 17.56% and 15.45% in *T. koningiopsis* TK alone. The RKN-infected soil had a lower abundance of 12.90% *Bacillus* sp. when compared to treated soil samples. The *Bacillus megaterium* in *B. velezensis* VB7 + *T. koningiopsis* TK drenched in RKN soil had a relative abundance of 12.45%. It was 10.34% in *B. velezensis* VB7 + RKN and 7.29% in *B. velezensis* VB7 alone applied soil. The lowest relative abundance of 4.78% was found in RKN-infested soil. *Pseudomonas barbori* was abundant in *T. koningiopsis* TK + *B. velezensis* VB7 + RKN soil at 9.19%. The *B. velezensis* VB7 + RKN soil had an abundance of *Pseudomonas barbori* of 8.01% against 5.21% in *T. koningiopsis* TK + RKN soil. The RKN soil had a lower abundance of only 1.75%, whereas *B. velezensis* VB7 alone comprised 6.5% against 3.4% in *T. koningiopsis* TK alone (Fig. 2F).

#### The Taxonomic Abundance of Bacterial Microbiome Population

**Fig. 3:**
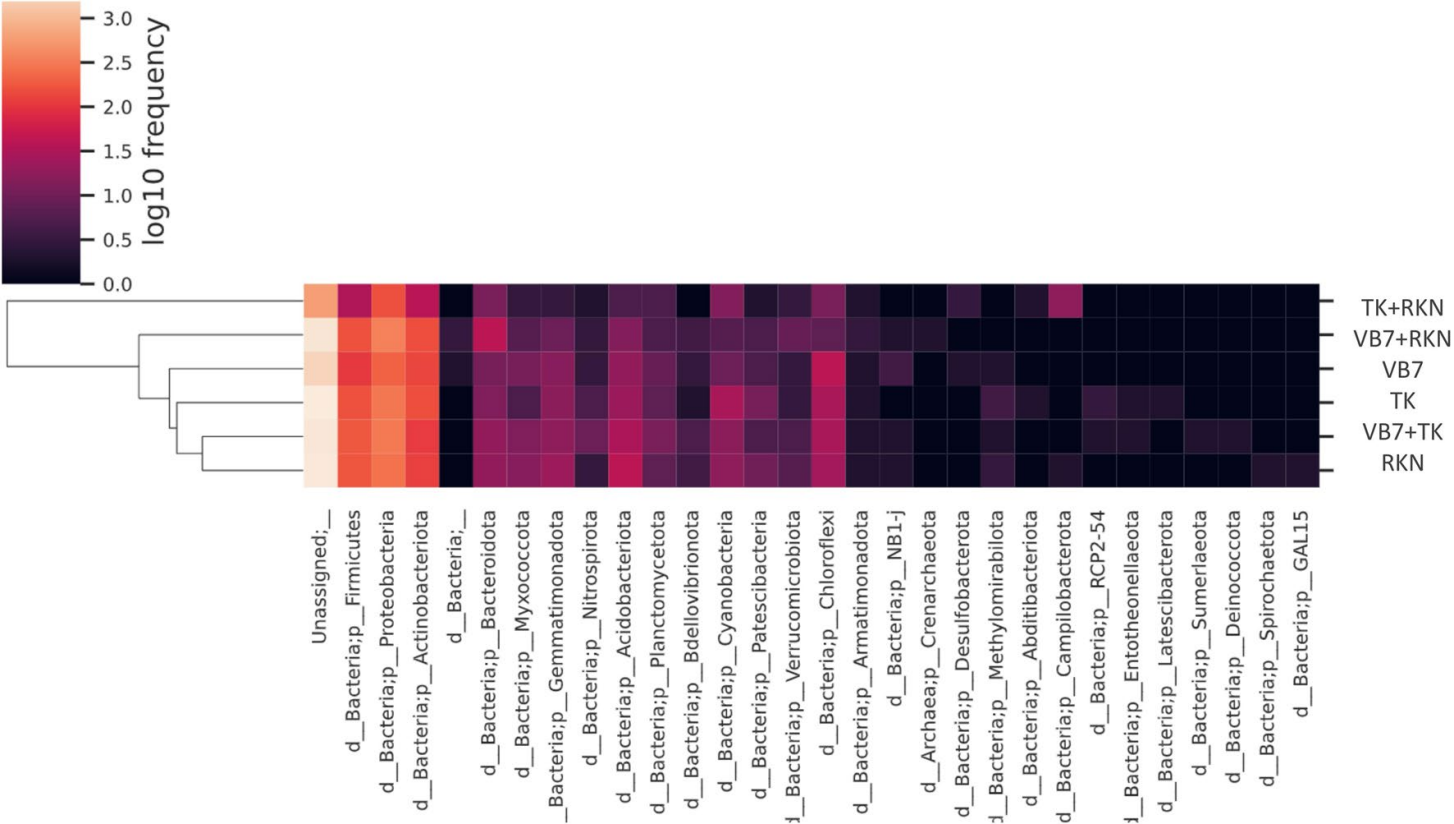
Cluster heatmap analysis of rhizosphere bacterial communities concerning different treatments at the phylum level (VB7 + RKN = *B. velezensis* VB7 + root-knot nematode, VB7 = *B. velezensis* VB7 alone, TK + RKN = *T. koningiopsis* TK + root-knot nematode, TK = *T. koningiopsis* TK alone, VB7 + TK + RKN = *B. velezensis* VB7 + *T. koningiopsis* TK + root-knot nematode; RKN, root-knot nematode (*M. incognita*))

Based on the information regarding microbial communities at the taxonomic level, a heatmap was constructed by clustering similar communities of each sample to determine the frequency of microbial communities. The phylum of Proteobacteria, Firmicutes, and Actinobacteria showed higher frequencies in all the samples, while Acidobacteriota, Cyanobacteria, and Chloroflexi had a lower frequency, including in the control (Fig. 3).

*Bacillus* and *Sphingomonas* had a relatively higher frequency than similar microbial communities in treated and untreated soil samples (Fig. S7). The higher prevalence of *Pseudomonas* was observed in the combined application of *B. velezensis* VB7 + *T. koningiopsis* TK + RKN treatment, whereas lower frequency was found in *T. koningiopsis* TK alone drenched soil. Compared to all other treatments, *Microbacterium*, *Ochrobacterium*, *Achromobacter*, and *Stenotrophomonas* were observed with a maximum frequency in *B. velezensis* VB7 + RKN soil. This bacterial genus had A comparatively lower frequency in untreated RKN-associated soil samples.

#### Comparison of OTUs at Different Treatments Using Venn Diagram

**Fig. 4:**
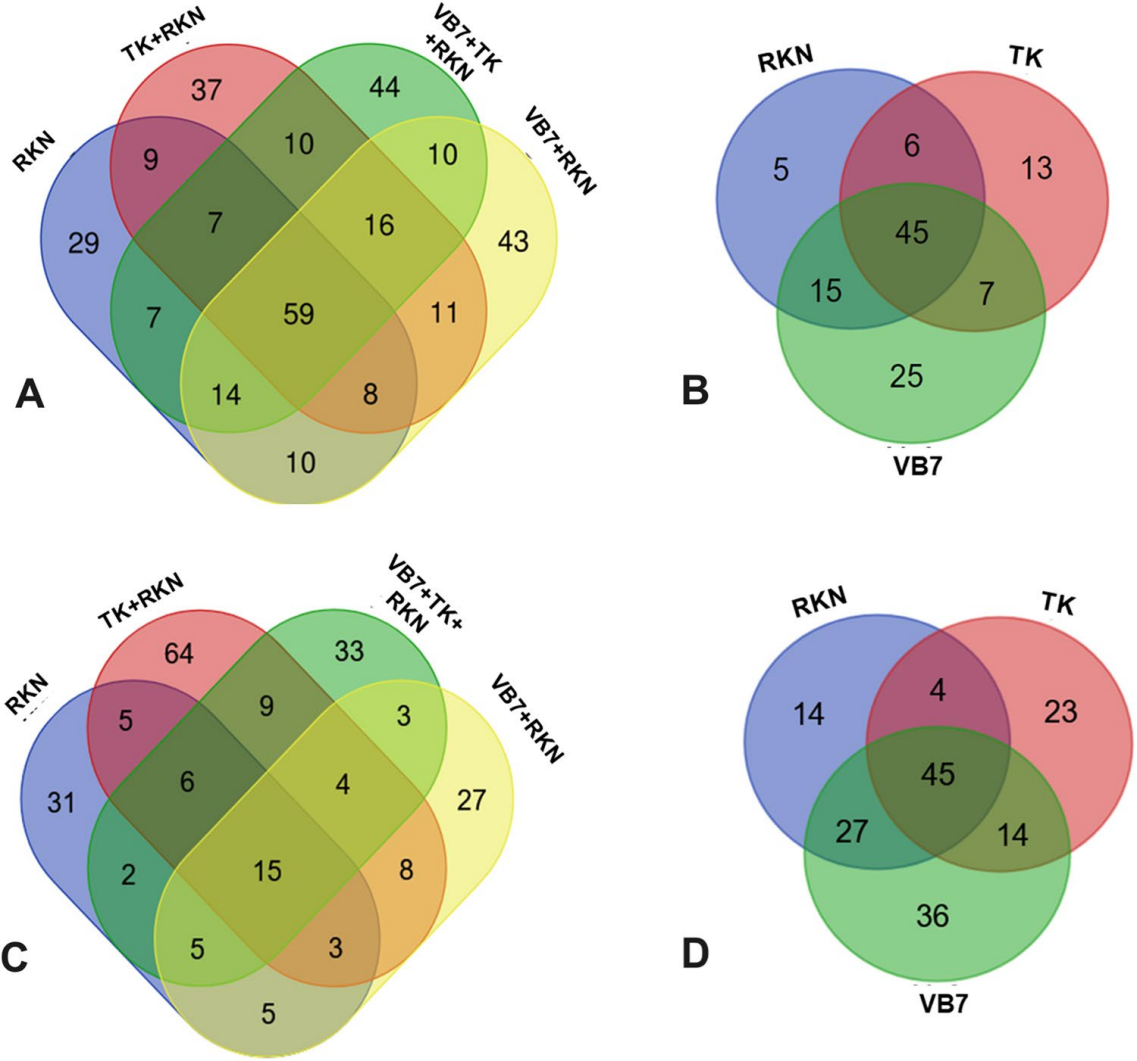
**A** Comparison of bacterial OTUs distributed in combined application (bioagents with fluazaindolozine) treatments at the genus level. **B** Comparison of bacterial OTUs distributed in the individual application (bioagents alone and biocontrol alone) treatments at genus level. **C** Comparison of bacterial OTUs distributed in the combined application (bioagents with fluazaindolozine) treatments at the species level. **D** Comparison of bacterial OTUs distributed in the individual application (bioagents alone and biocontrol alone) treatments at species level (VB7 + RKN = *B. velezensis* VB7 + root knot-nematode, VB7 = *B. velezensis* VB7 alone, TK + RKN = *T. koningiopsis* TK + root-knot nematode, TK = *T. koningiopsis* TK alone, VB7 + TK + RKN = *B. velezensis* VB7 + *T. koningiopsis* TK + root-knot nematode; RKN, root-knot nematode (*M. incognita*))

A total of 3788 OTUs were obtained from the high-throughput sequencing. The OTUs distribution in the combined applications, i.e., *B. velezensis* VB7 + RKN, *T. koningiopsis* TK + RKN, *B. velezensis* VB7 + *T. koningiopsis* TK + RKN, and control (untreated) soil samples were compared at genus and species levels to determine similar organisms shared between treatments. At the genus level, a total of 59 OTUs were shared by all four soil treatments. Among them, 29 OTUs were found in untreated RKN control, 37 OTUs were shared in *T. koningiopsis* TK + RKN, *B. velezensis* VB7 + RKN had 44 OTUs, and *B. velezensis* VB7 + *T. koningiopsis* TK + RKN soil constituted of 43 OTUs, respectively (Fig. 4A). Fifteen OTUs were common for all four of the above-mentioned soil treatments at the species level, with unique OTUs of 27, 33, 31, and 64, respectively (Fig. 4B).

Similarly, the OTU distribution in the individual applications, i.e., *B. velezensis* VB7 alone, *T. koningiopsis* TK alone, and control (untreated) soil samples, was compared at the genus and species levels. A total of 45 OTUs at the genus level and species level were commonly distributed in all combined application treatments, followed by similar OTUs of 25, 13, and 5 at the genus level and 36, 23, and 16 OTUs at the species level for *B. velezensis* VB7 alone, *T. koningiopsis* TK alone and control (untreated) soils (Fig. 4C and D).

### Diversity of Bacterial Communities in Different Rhizosphere Soils

#### Alpha Diversity Indexes

**Fig. 5:**
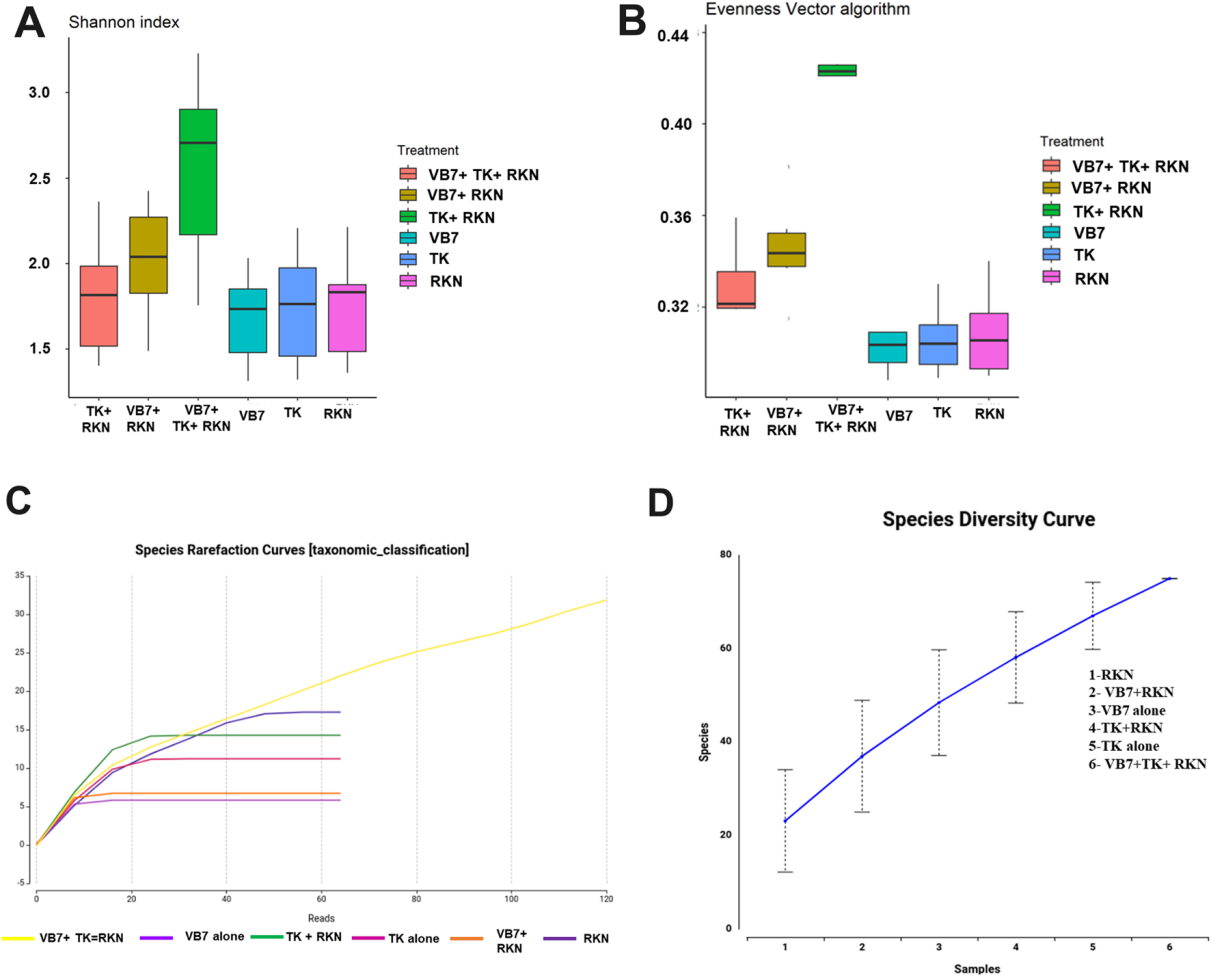
Alpha diversity index for rhizosphere bacterial communities distributed with respect to different treatments. **A** Shannon index. **B** Evenness Vector Algorithm. **C** Refraction Curve. **D** Species Diversity Curve (VB7 + RKN = *B. velezensis* VB7 + root-knot nematode, VB7 = *B. velezensis* VB7 alone, TK + RKN = *T. koningiopsis* TK + root-knot nematode, TK = *T. koningiopsis* TK alone, VB7 + TK + RKN = *B. velezensis* VB7 + *T. koningiopsis* TK + root-knot nematode; RKN, root-knot nematode (*M. incognita*))

Alpha diversity is applied to analyze the richness and diversity of microbial communities present in the soil. The rhizosphere soil of *B. velezensis* VB7 + *T. koningiopsis* TK + RKN had the maximum Shannon index in all taxonomic levels from phylum to species with the range of 1.57 to 2.90, followed by *B. velezensis* VB7 + RKN (1.87 to 2.32). The bacterial communities in RKN-infested soil and *T. koningiopsis* TK-drenched soil had almost similar levels of richness with lower indexes than other samples (Fig. 5A). The evenness vector algorithm indicated that tomato rhizosphere soil drenched with *B. velezensis* VB7 + *T. koningiopsis* TK + RKN contained higher species diversity with more bacterial community richness than other treatments. *T. koningiopsis* TK, *T. koningiopsis* TK + RKN, and RKN-infected soil samples had similar levels of bacterial species (evenness) with greater richness except for RKN soil, which had a higher level of microbial diversity with lesser homogeneity of organisms observed (Fig. 5B). The refraction curve showed a significant increase in bacterial species, indicating that nematicide-treated soil had the maximum bacterial communities with more diverse bacterial species compared to other soil treatments (Fig. 5C). The Species Diversity Curve analysis showed that the number of bacterial species in the soil varied according to each treatment. The RKN-infested tomato rhizosphere soil treated with the combined form of *B. velezensis* VB7 + *T. koningiopsis* TK had greater species diversity when compared to *T. koningiopsis* TK, *B. velezensis* VB7 treated soil without RKN. At the same time, lower species diversity was observed in untreated RKN-infected soil (Fig. 5D).

#### Beta Diversity Indexes

**Fig. 6:**
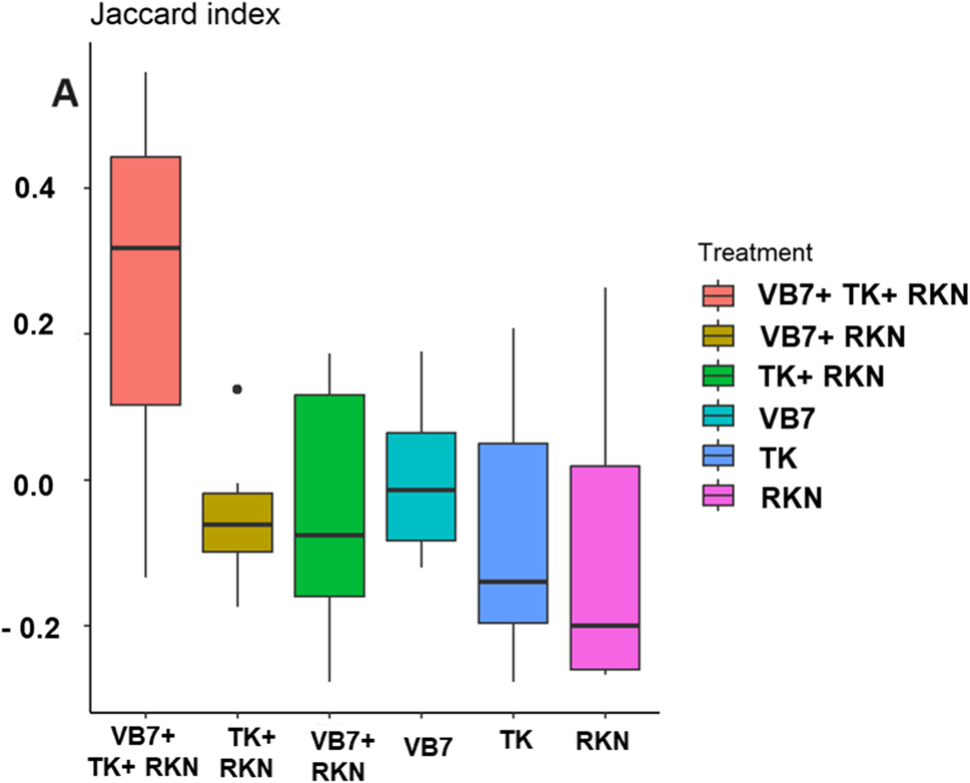
Beta diversity index for rhizosphere bacterial communities distributed with respect to different treatments. **A** Jaccard Index (VB7 + RKN = *B. velezensis* VB7 + root-knot nematode, VB7 = *B. velezensis* VB7 alone, TK + RKN = *T. koningiopsis* TK + root-knot nematode, TK = *T. koningiopsis* TK alone, VB7 + TK + RKN = *B. velezensis* VB7 + *T. koningiopsis* TK + root-knot nematode; RKN, root-knot nematode (*M. incognita*))

The beta diversity was measured using the Bray Curtis algorithm and Jaccard algorithm index. The Bray Curtis algorithm showed that the *B. velezensis* VB7 + *T. koningiopsis* TK + RKN had substantially different communities (0.15), whereas other samples exhibited 99% similar bacterial community compositions. The values of the Jaccard algorithm index indicated that the diversity and similarity between the organisms varied for each sample at every taxonomic level. The bacterial communities in the rhizosphere soil had greater diversity with lesser homogeneity between populations (Fig. 6A).

#### Co-occurrence Clustering Coefficient Analysis of Bacterial Communities in Treated and Untreated Soil Samples

**Fig. 7:**
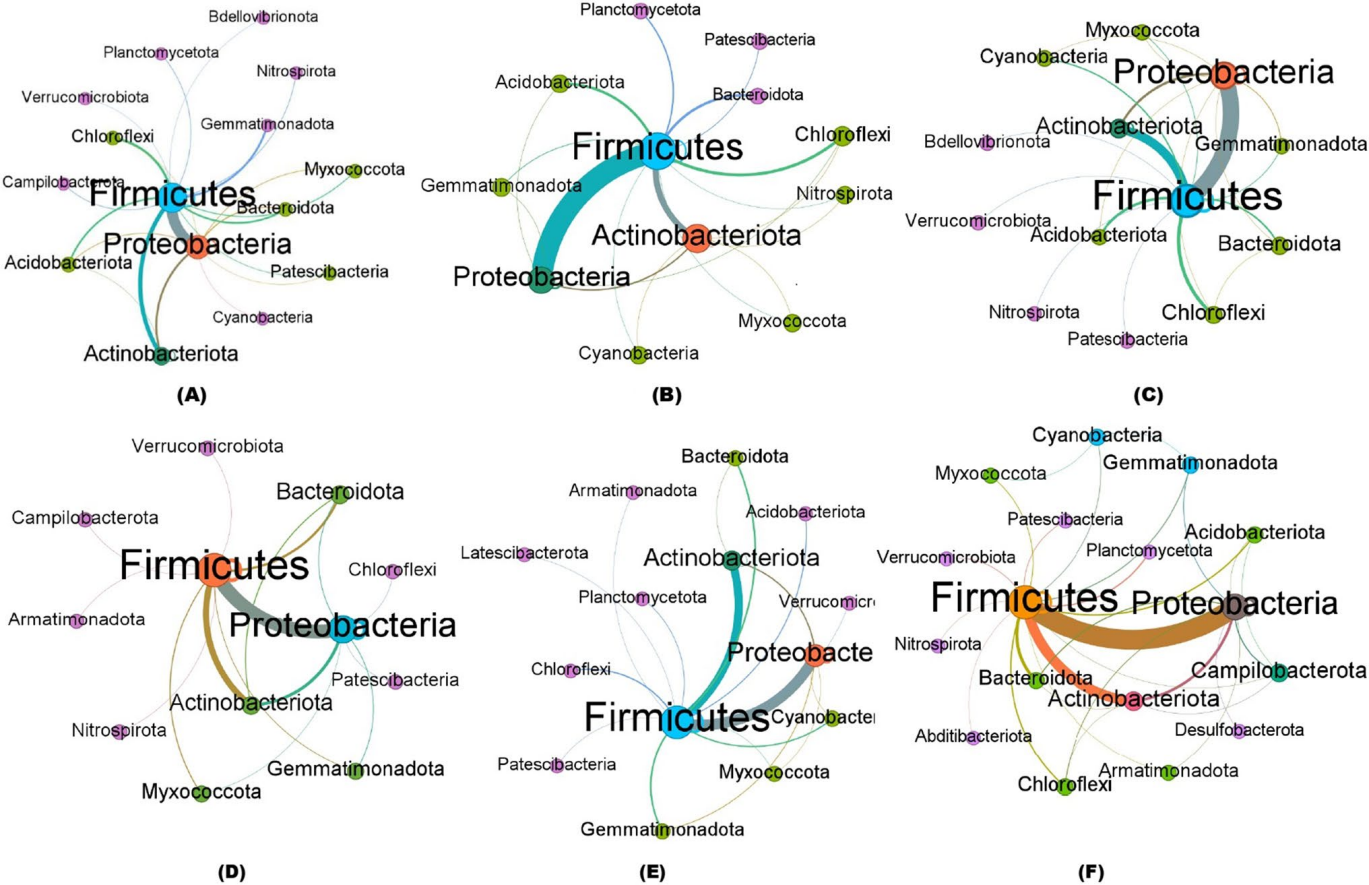
Co-occurrence clustering coefficient networks of bacterial communities obtained from treated and RKN-infected soil. **A** Co-occurrence clustering coefficient network of different bacterial phyla present in RKN-infected soil alone. **B** Co-occurrence clustering coefficient network of different bacterial phyla present in the *T. koningiopsis* TK alone applied soil. **C** Co-occurrence clustering coefficient network of different bacterial phyla in *B. velezensis* VB7 alone treated soil. **D** Co-occurrence clustering coefficient network of different bacterial phyla present in *T. koningiopsis* TK treated soil with the association of RKN. **E** Co-occurrence clustering coefficient network of different bacterial phyla in *B. velezensis* VB7 treated soil with the association of RKN. **F** Co-occurrence clustering coefficient network of different bacterial phyla in *B. velezensis* VB7 + *T. koningiopsis* TK with RKN associated soil. Each node represents a bacterial phylum, whereas the edges represent a clustering coefficient, with a magnitude of 0.01 to 1.00 between the nodes. Each node is labeled at the phylum level. The thickness of the edges represents the strength of clustering and interaction of bacterial species. The thicker edges, with a boldness of bacterial phylum, had a more significant clustering and strong interaction between bacterial communities. The green color nodes and edges represent the interaction and co-occurrence of Proteobacteria; orange color nodes and edges represent the interaction and co-occurrence of firmicutes; brown color nodes and edges represent the interaction and co-occurrence of Actinobacteria

The co-occurrence patterns of all networks differed significantly among treated and untreated RKN-infected soil samples. Co-occurrence network analysis revealed similar nodes (phylum) among the bacteria in the soil communities obtained from the biocontrol agents and nematicide-treated and untreated soil samples (Fig. 7A–F). However, the numbers of nodes and their interconnecting edges (lines) differed. The bacterial phyla, Proteobacteria, Firmicutes, and Actinobacteria, were clustered in more significant proportions with strong interactions (thicker edges with bold letters—greater occurrence) in all the soil samples. The nodes of Proteobacteria interconnecting with other bacterial edges are highlighted in green, whereas the interconnection of Firmicutes is represented in orange (nodes and edges), while brown-colored nodes and edges indicate the population of Actinobacteria. The strong correlation between the abundance of bacterial phylum (thicker lines) with a higher number of interconnection edges was more significant in Proteobacteria, Firmicutes, and Actinobacteria phyla in RKN-associated soils treated with *B. velezensis* VB7 + *T. koningiopsis* TK (Fig. 7F). The interaction in *B. velezensis* VB7 with RKN-associated soil had a diverse effect with higher interactions than *B. velezensis* VB7 alone without RKN (Fig. 7D and E). The *T. koningiopsis* TK + TKN and *T. koningiopsis* TK alone treated soils had sparse edges with a lower coefficient and stronger interactions than RKN samples (Fig. 7B and C). The untreated control RKN soil had diverse, with thinner interconnecting edges (more inferior) noticed between bacterial communities (Fig. 7A). Collectively, the data provided further evidence that the bacterial community in tomato rhizosphere soil was increased in the presence of the combined application of *B. velezensis* VB7 or *T. koningiopsis* TK, exhibiting a significant increase in the relative abundance of bacterial communities due to synergistic interactions.

## Discussion

Recently, plant pathogenic nematodes have emerged as a major yield-limiting factor in vegetable crops across the globe. At this juncture, the reduction of plant parasitic nematodes depends on the interaction of different biotic and abiotic factors in the soil. In our investigation, *T. koningiopsis* TK was highly compatible with *B. velezensis* VB7. Combined application of *B. velezensis* VB7 + *T. koningiopsis* TK had the highest nematicide activity on the hatching of eggs and its mortality compared to individual application of *B. velezensis* VB7, *T. koningiopsis* TK, and the untreated control. Consistent with these findings, Tian et al. [38] reported that, within 12 h of exposure to the secondary metabolites of *B. velezensis*-25, 100% mortality of juveniles of *M. incognita* was observed. Similarly, the cultural filtrates of *B. velezensis* CE and *B. thuringiensis* KYC had the lowest egg-hatching rates of 2.5% and 9% at 40% concentration, respectively [39]. *B. amyloliquefaciens* had the highest level of suppression in egg hatching and induced the mortality of *M. incognita* J2s, besides promoting plant growth [40]. According to Sreenayana et al. [41], the conidial suspension of *T. koningiopsis* TRI 41 effectively reduced the egg hatching of RKN up to 71.51% and 73.0% of juvenile mortality in cucumber. These findings agreed with our findings that combining *B. velezensis* VB7 and *T. koningiopsis* TK reduced juvenile mortality and inhibited the egg-hatching of root-knot nematode in tomatoes.

Hence, we conducted a field trial for RKN infection in tomatoes in an endemic location through the combined application of *B. velezensis* VB7 and *T. koningiopsis* TK. Metagenomic analysis was carried out from the field samples to investigate the diversity and richness of bacterial communities in the rhizosphere region. Microbiome investigations have targeted bacterial and fungal biomes that are phylogenetically well-characterized and were strongly associated with plant health. It reflects the relative abundance and biodiversity of the microbiome in the rhizosphere [42]. Tomato plant roots are strongly related to diverse microbial communities in the soil [43], which have a unique role in plant growth and development. Several investigations on rhizomicrobiome emphasized the presence of diverse microbes in the root zone, thus contributing towards the enhanced antagonistic efficacy against PPN in vegetable crops [44]. Combining beneficial microorganisms can effectively induce defense mechanisms against PPN [45]. Based on the scientific evidence by Zaim and his coworkers [46], we investigated the compatibility of *Trichoderma* spp. with *Bacillus* spp. to determine the synergistic effect of biocontrol agents to promote the microbiome’s diversity. In addition, several previous investigations have also confirmed that parasitism by RKN had a positive correlation with the richness and diversity of microbial communities associated with the suppression of RKN [14. 49–52].

The present study aimed to apply effective BCA, *B. velezensis* VB7, and *T. koningiopsis* TK against RKN infestation in tomato rhizosphere. Maintaining high population densities of these microorganisms after inoculation is a substantial constraint since they deteriorate with time and even at a distance from the source of the inoculum. Hence, we conducted the present study to assess the variations in microbial diversity in the rhizosphere region by the combined application of *B. velezensis* VB7 and *T. koningiopsis* TK and through the individual application of bioagents on tomato plants in the soil endemic to RKN infestation. The composition relative abundance of the phyla Proteobacteria (42.16%), Firmicutes (19.57%), and Actinobacteriota (17.69%) being dominant in the soil samples drenched with the combined application of *B. velezensis* VB7 and *T. koningiopsis* TK. Our findings were consistent with evidence from numerous reports [22, 51]. The high abundance of Proteobacteria in the rhizosphere largely reflects the abundance of γ-proteobacteria, followed by α-proteobacteria. Hitherto, the microbial consortia can substantially modify the composition of the bacterial rhizosphere community by promoting the establishment and development of beneficial bacteria. Rhizobiales were frequently noticed in the rhizosphere of healthy tomato plants and reduced richness and abundance in the bacterial rhizobiome. The reduction in Rhizobiale diversity was attributed to a change in the composition and richness of microbiota during nematode infection. Tian and coworkers [29] reported changes in the abundance of the dominant species of Rhizobiales in the root microbiome during the infestation of RKN in crop plants.

In particular, the genus *Bacillus* was dominant in all rhizosphere soil samples. However, the relative abundance was highest in the RKN-associated soils treated with a combined application of *B. velezensis* VB7 + *T. koningiopsis* TK. *Bacillus* spp. were ubiquitous in the rhizosphere; consequently, these bacteria suppressed the interaction of RKN with tomato roots and thus acted as a bio-nematicide [52]. Rhizobacteria, such as *Bacillus*, may positively influence tomato plant growth, functioning as biofertilizers to increase tomato growth [53]. On the other hand, *Bacillus* was dominant in the presence of RKN, while *Cellvibrio* and *Steroidobacter* were more abundant in RKN-infested soils drenched with nematicide [54].

The drenching of RKN-associated rhizosphere with *B. velezensis* VB7 also increased the abundance frequency of *Bacillus*, *Sphingomonas*, and *Pseudomonas*, while *Microbacterium Achromobacter* and *Ochrobactrum* were more abundant in *B. velezensis* VB7 in the presence of RKN, as compared to Marinomonas and Stenotrophomonas in *T. koningiopsis* TK, treated soil. In agreement with these findings, the work of Kour et al. [55] confirmed the presence of rhizospheric microbes, including *Acinetobacter*, *Achromobacter*, *Bacillus*, *Burkholderia*, *Flavobacterium*, *Micrococcus*, and *Pseudomonas*, has a positive impact on plant growth in RKN-infested soil samples. *B. velezensis* Bv-25 had significant nematicidal activity against *M. incognita*, significantly reducing the severity of *M. incognita* and increasing the cucumber yield under field conditions [38]. Similarly, the bacterial genera *Sphingomonas*, *Micrococcus*, *Bacillus*, *Methylobacterium*, *Rhizobium*, and *Bosea* were dominant in soils suppressing *M. hapla* [56]. In agreement with earlier findings of many researchers in discussion [22, 38–56], our work detected an increase in the relative abundance of *Bacillus* spp., Firmicutes, *Sphingomonas* spp., and *Pseudomonas* spp. in soils drenched with *B. velezensis* VB7 and *T. koningiopsis* TK in RKN infested soil. Thus, the synergistic interaction and compatibility of biocontrol agents might have triggered defense mechanisms, plant growth promotion, and yield, as suggested by earlier researchers [46].

The dominance of *Vicinamibacteria* and *Microvirga* was also observed in soils without RKN infestation drenched with *B. velezensis* VB7 alone. The added biocontrol agents might have served as signals to increase the microbiome’s diversity and abundance level.

The tomato rhizosphere soils drenched with biogents of *T. koningiopsis* TK and *B. velezensis* VB7 increased the relative abundance of *Pseudomonas*, *Marinomonas*, *Microvirga*, and *Marinomonas* might have reduced the RKN infestation. The microbes associated with the suppression of RKN pertain to *Acinetobacter*, *Bacillus*, *Enterobacter*, *Microbacterium*, *Paenibacillus*, *Pseudomonas*, and *Streptomyces* [57].

The *Pseudomonas* genus from the Pseudomonaceae family and the *Marinomonas* genus from the Oceanospirillaceae family were also observed abundantly in tomato rhizosphere soil drenched with *T. koningiopsis* TK in RKN-infested soil, compared to untreated RKN-infested soil samples. An increased abundance of γ-proteobacteria, including *Pseudomonas* species, produces antimicrobial compounds responsible for suppressing RKN [58]. Certain *Pseudomonas* strains’ role in suppressing *M. javanica* in tomato plants concludes that Induced Systemic Resistance (ISR) was the primary mechanism for reducing nematode infestation [59]. The relative abundance of bacterial genera *Cellvibrio*, *Microbacterium*, *Gaiella*, and *Nocardioides* was abundantly distributed and proliferated more in individual applications of *B. velezensis* VB7 and *T. koningiopsis* TK without RKN than in untreated RKN infested soil samples. The aforementioned genera were previously identified in cumber rhizosphere soil and shown to stimulate plant development and decrease plant diseases [60]. Similarly, in the present study, an increase in the abundance of the above bacterial community might have also contributed to plant growth and reduced RKN infestation.

In the present study, Alpha and Beta diversity indexes indicated a higher microbiome abundance and diversity in treated than in untreated soils, which agreed with the findings of Tian et al*.* [29]. The reduction in the severity of RKN infestation has been attributed to the combined actions of several biocontrol agents [61–63]. In concert with this data, our finding also indicated that *Pseudomonas* spp. was abundant in plant roots treated with *B. velezensis* VB7, both with and without RKN.

Several investigations have confirmed that *Trichoderma* spp. inhabits and colonizes plant roots, enhancing their defensive mechanisms against nematodes. The induction of defense against RKN by *Trichoderma* spp. is also mediated through JA and SA pathways [64–66]. In addition, biotic pressure on the root microbial community increased the microbial diversity under the persistent disease state, which resulted in an increase in some minor taxa and a reduction of the dominant core species [67]. The increased interaction between Proteobacteria, Firmicutes, Actinobacteria, and other bacteria in the rhizosphere bacterial network defends against RKN infestation in tomatoes. The increased relative abundance of beneficial bacterial taxa, especially Proteobacteria, in the rhizosphere of microbial consortia-treated soil plots and the higher abundance of genes associated with the indicated cellular processes influenced tuber yield in potatoes [68].

Hence, the diverse community of microorganisms that inhabit the soil around plant roots can play an essential role in helping the plants defend themselves against plant-parasitic nematodes. The diversity of microbial communities has also facilitated metabolic interaction to execute intricate functions that an individual organism might have accomplished. Members of the consortia or communities may interact by exchanging metabolites or molecular signals to regulate their activity through the temporal and geographical expression of essential processes [69]. Using genomic approaches, the four best-suited genera for developing biocontrol inoculants are *Bacillus*, *Chitinophaga*, *Rhizobium*, and *Burkholderia* [70]. This suggests that changing the rhizosphere microbiome may positively influence plants to tolerate RKN infestation.

The biocontrol agents immediately control the nematodes and other pathogens and reduce the risk of developing resistance [71–78]. Further, compatibility between *B. velezensis* VB7 and *T. koningiopsis* TK also increased the relative abundance of the beneficial bacterial community in the tomato rhizosphere, which might have complimented the multiple modes of action to suppress RKN in the tomato rhizosphere.

## Conclusion

We investigated the effects of microbial diversity from biocontrol agents and nematicide-treated tomato plants through high-throughput sequencing analysis. Our results revealed that treated and untreated soil had a larger microbial community abundance. The bacterial phylum Proteobacteria, followed by firmicutes and actinobacteria, were dominant. The soil treated with biocontrol agents had the maximum diversity and relative abundance with biologically active microbial genera. *Bacillus*, *Pseudomonas*, and *Sphingomonas* were bionematicides against RKN and enhanced plant growth. Overall, the study suggested that manipulating the bacterial microbiome in the rhizosphere through the use of nematicidal molecules along with microbial biocontrol agents could be an effective strategy for managing plant nematode infections, thereby improving plant growth and yield.

## Supplementary Information

Below is the link to the electronic supplementary material.

Fig. S1 Composition of bacterial phyla in rhizosphere soil concerning different treatments (PNG 478 kb)

High resolution image (TIF 426 kb)

Fig. S2 Composition of bacterial classes in rhizosphere soil concerning different treatments (PNG 538 kb)

High resolution image (TIF 453 kb)

Fig. S3 Composition of bacterial orders in rhizosphere soil concerning different treatments (PNG 600 kb)

High resolution image (TIF 493 kb)

Fig. S4 Composition of bacterial families in rhizosphere soil concerning different treatments (PNG 607 kb)

High resolution image (TIF 529 kb)

Fig. S5 Composition of bacterial genera in rhizosphere soil concerning different treatments (PNG 564 kb)

High resolution image (TIF 460 kb)

Fig. S6 Composition of bacterial species in rhizosphere soil concerning different treatments (PNG 701 kb)

High resolution image (TIF 589 kb)

Fig. S7 Cluster heatmap for distribution of bacterial species in different treatments (Mg1 - *B. velezensis* VB7 + Root Knot Nematode, Mg2- *B. velezensis* VB7 alone, Mg3- *T. koningiopsis* TK + Root Knot Nematode, Mg4 - *T. koningiopsis* TK alone, Mg5 - *B. velezensis* VB7 + *T. koningiopsis* TK + Root Knot Nematode, Mg6 - Root Knot Nematode (*M. incognita*)) (JPG 6558 kb)

## Data Availability

All the data is included in the manuscript and supplementary files.
