# Supplementary figures and images for: Rhizosphere Engineering of Biocontrol Agents Enriches Soil Microbial Diversity and Effectively Controls Root-Knot Nematodes
Source: Microb Ecol. 2024 Sep 28;87(1):120. doi: 10.1007/s00248-024-02435-7 (PMC11438712; doi:10.1007/s00248-024-02435-7)

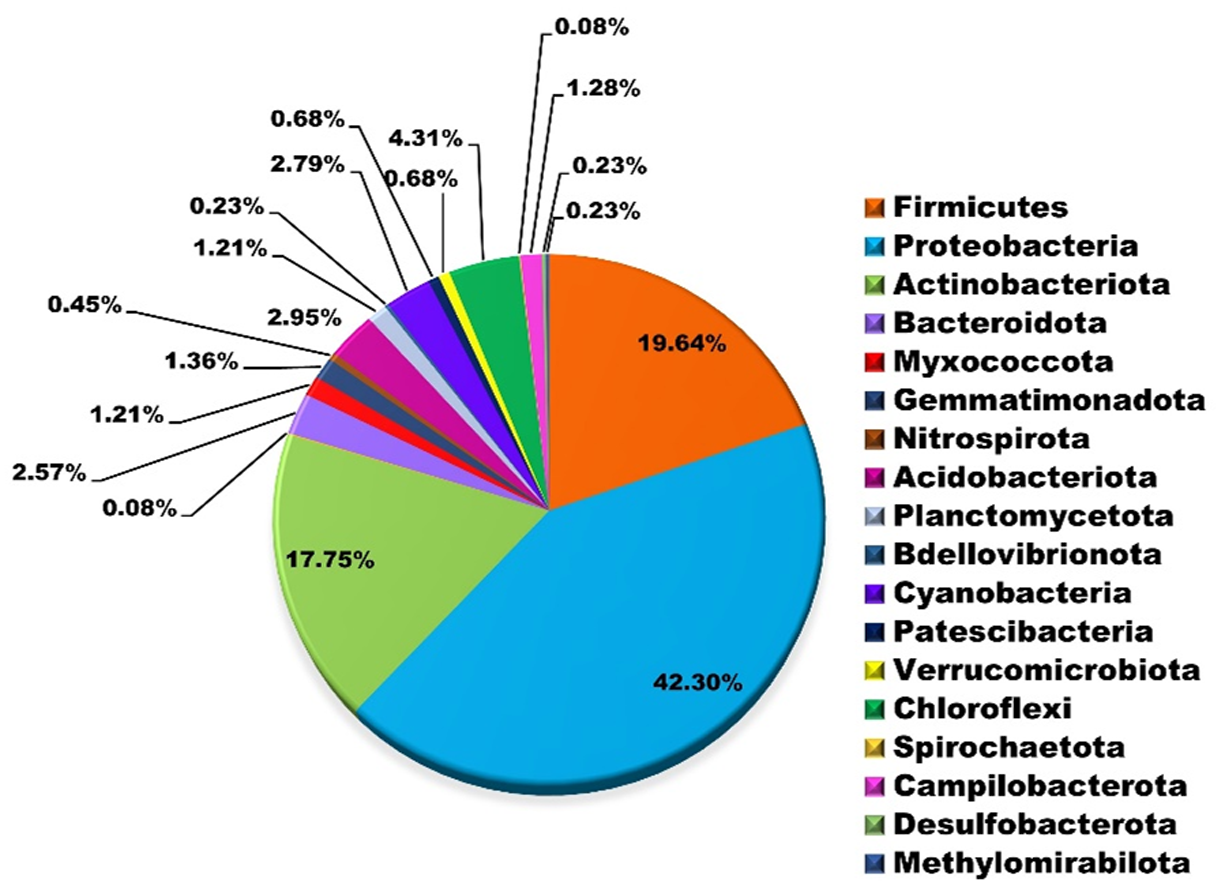

Supplement: Supplementary file 1 — Composition of bacterial phyla in rhizosphere soil concerning different treatments (PNG 478 kb) [file 248_2024_2435_Fig8_ESM.png]

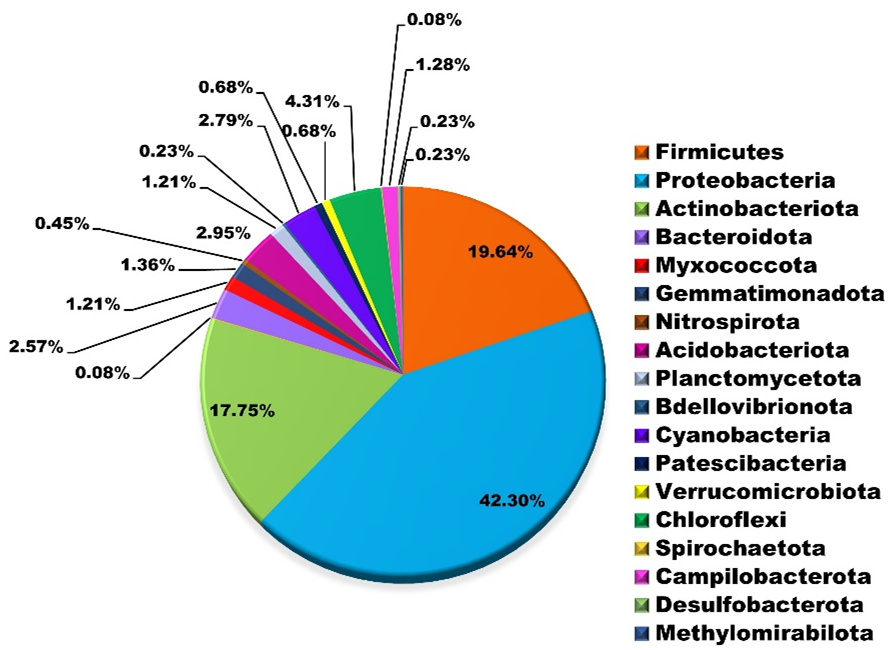

Supplement: Supplementary file 2 — High resolution image (TIF 426 kb) [file 248_2024_2435_MOESM1_ESM.tif]

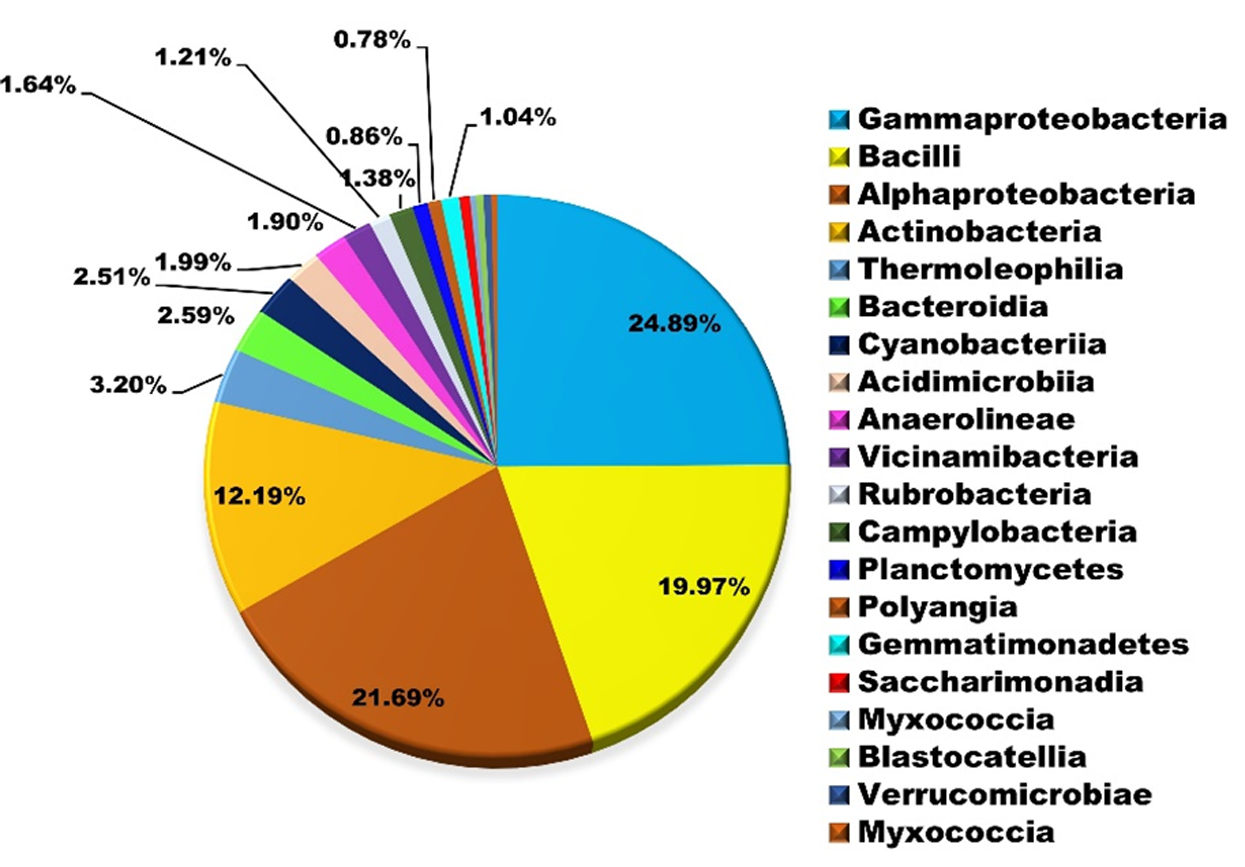

Supplement: Supplementary file 3 — Composition of bacterial classes in rhizosphere soil concerning different treatments (PNG 538 kb) [file 248_2024_2435_Fig9_ESM.png]

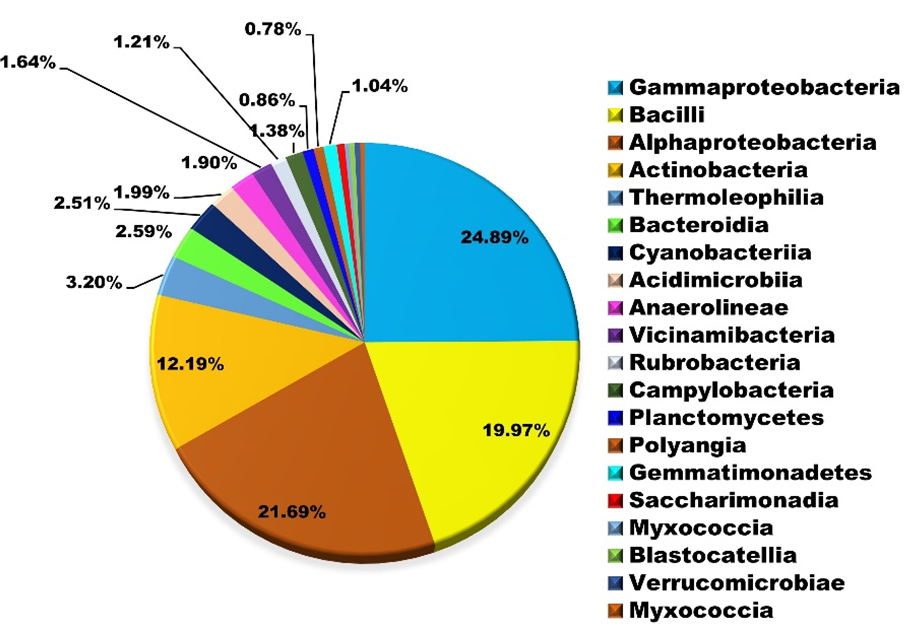

Supplement: Supplementary file 4 — High resolution image (TIF 453 kb) [file 248_2024_2435_MOESM2_ESM.tif]

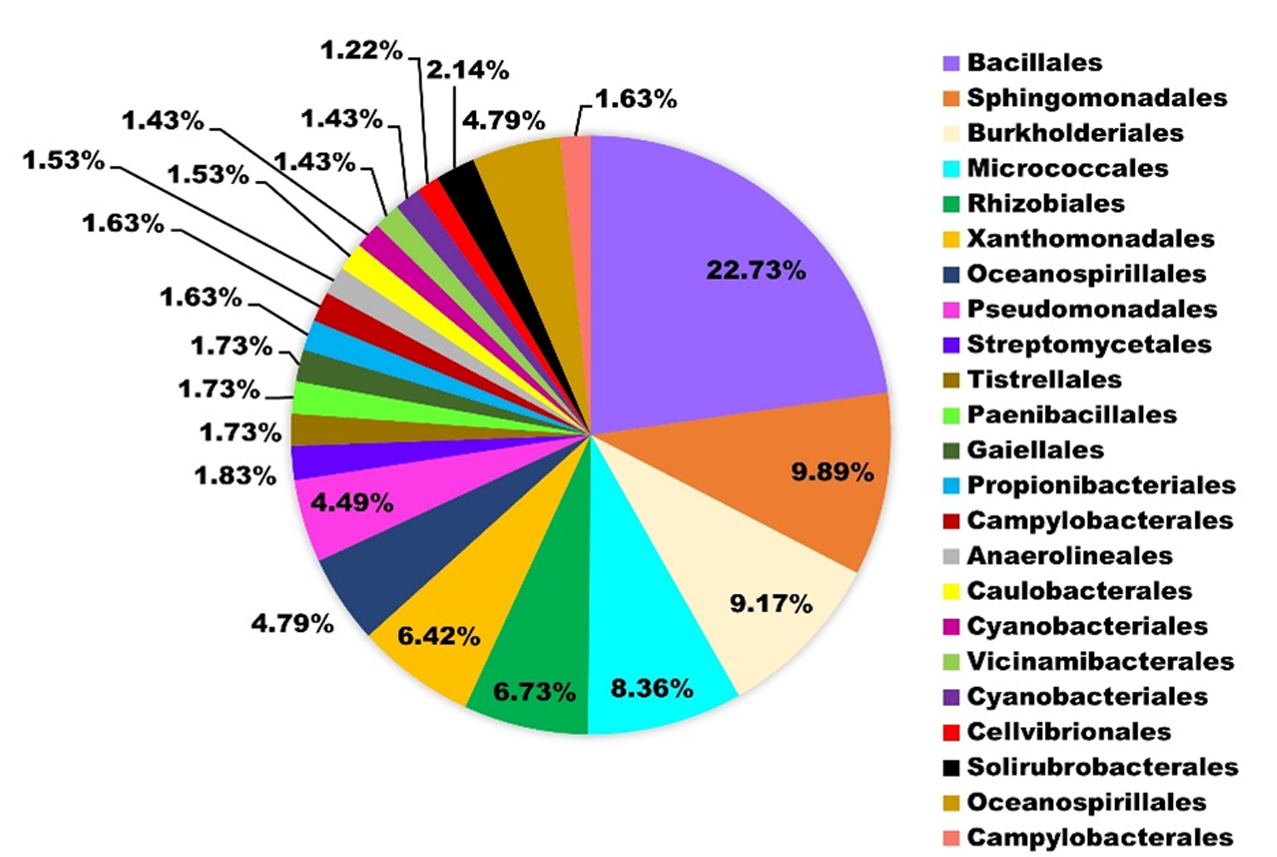

Supplement: Supplementary file 5 — Composition of bacterial orders in rhizosphere soil concerning different treatments (PNG 600 kb) [file 248_2024_2435_Fig10_ESM.png]

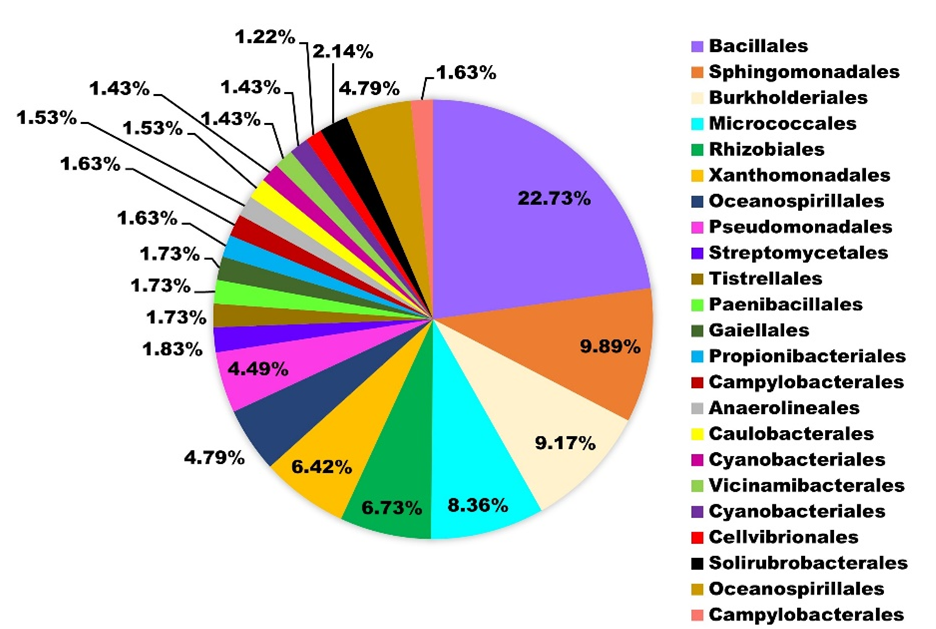

Supplement: Supplementary file 6 — High resolution image (TIF 493 kb) [file 248_2024_2435_MOESM3_ESM.tif]

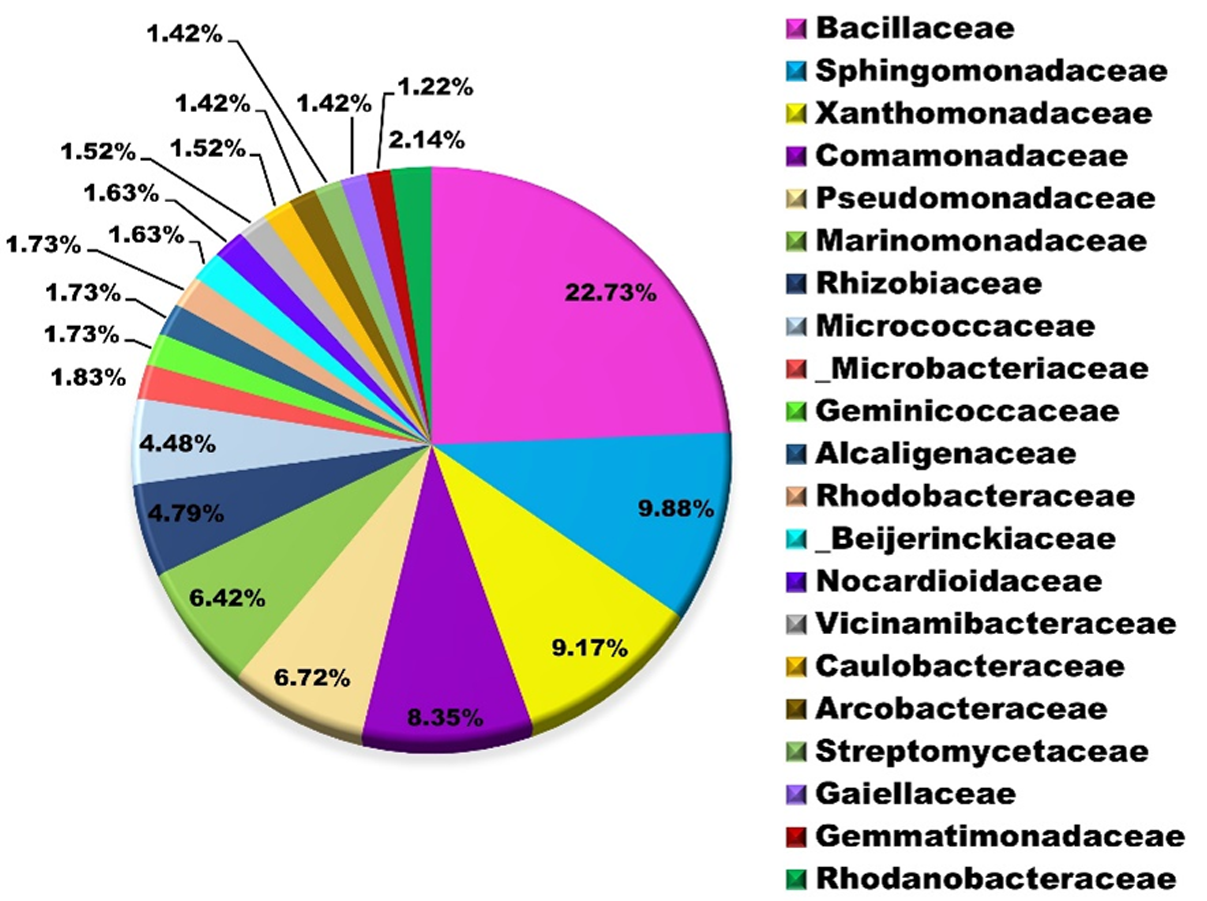

Supplement: Supplementary file 7 — Composition of bacterial families in rhizosphere soil concerning different treatments (PNG 607 kb) [file 248_2024_2435_Fig11_ESM.png]

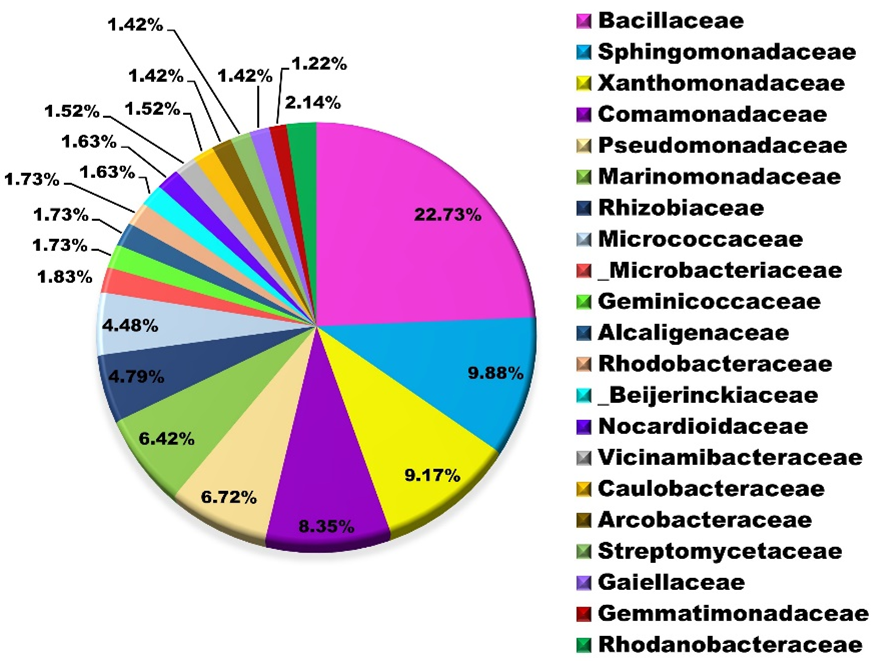

Supplement: Supplementary file 8 — High resolution image (TIF 529 kb) [file 248_2024_2435_MOESM4_ESM.tif]

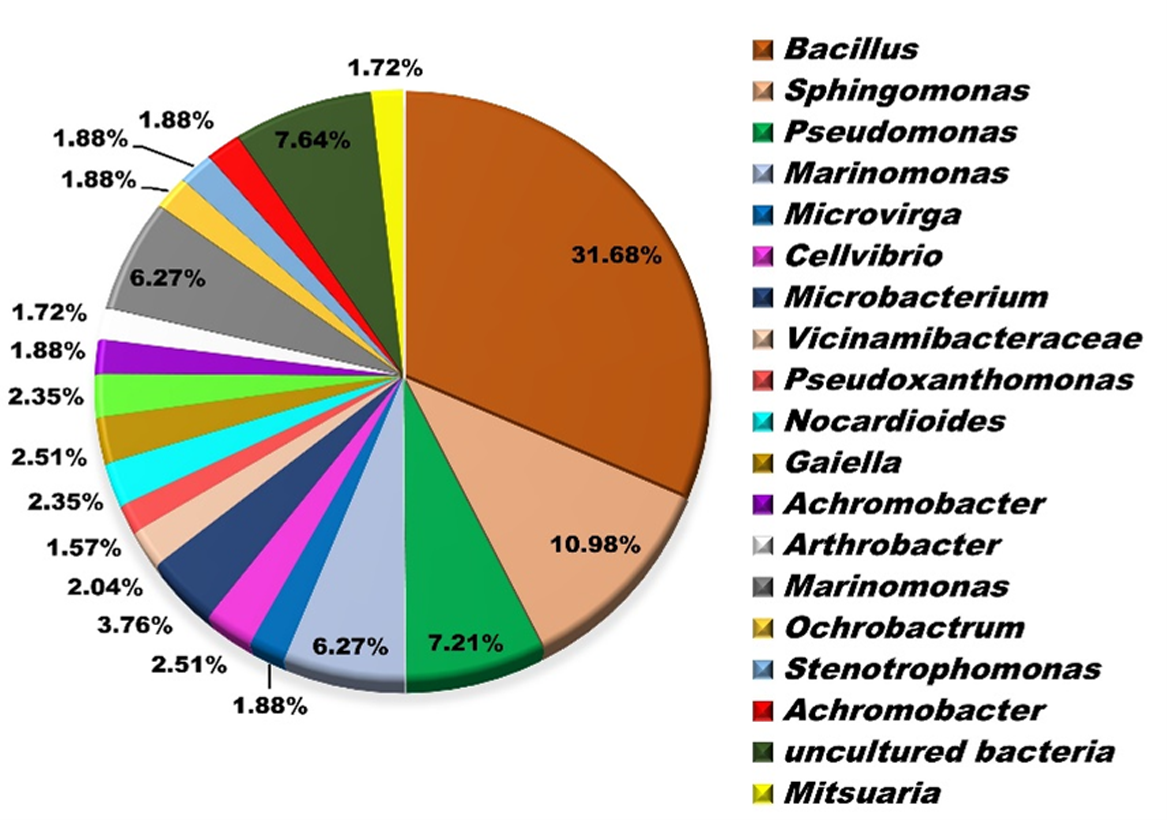

Supplement: Supplementary file 9 — Composition of bacterial genera in rhizosphere soil concerning different treatments (PNG 564 kb) [file 248_2024_2435_Fig12_ESM.png]

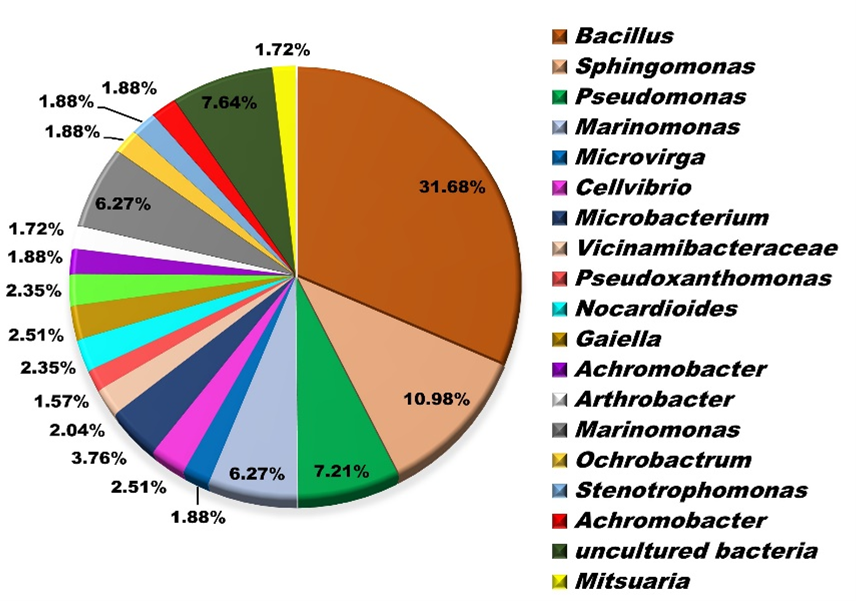

Supplement: Supplementary file 10 — High resolution image (TIF 460 kb) [file 248_2024_2435_MOESM5_ESM.tif]

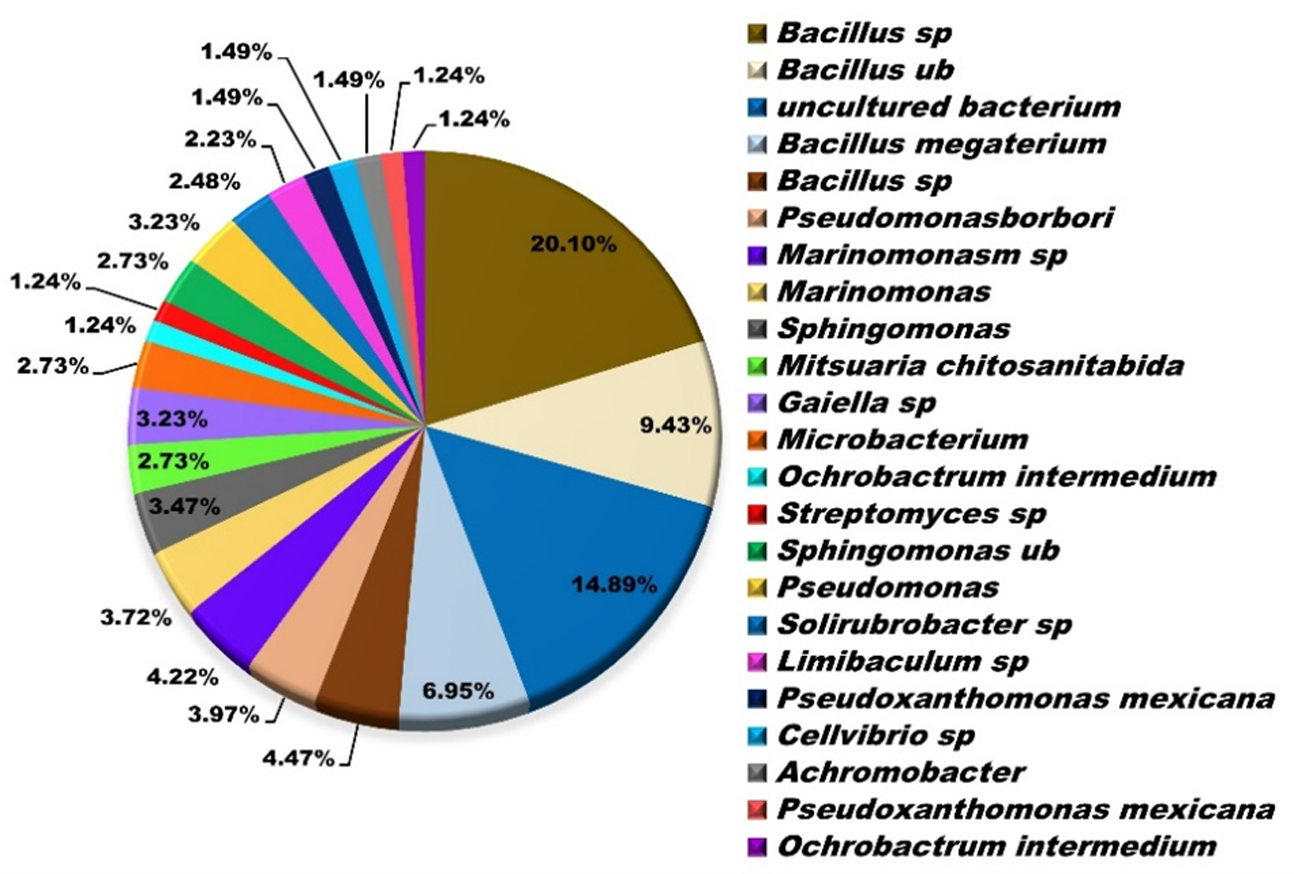

Supplement: Supplementary file 11 — Composition of bacterial species in rhizosphere soil concerning different treatments (PNG 701 kb) [file 248_2024_2435_Fig13_ESM.png]

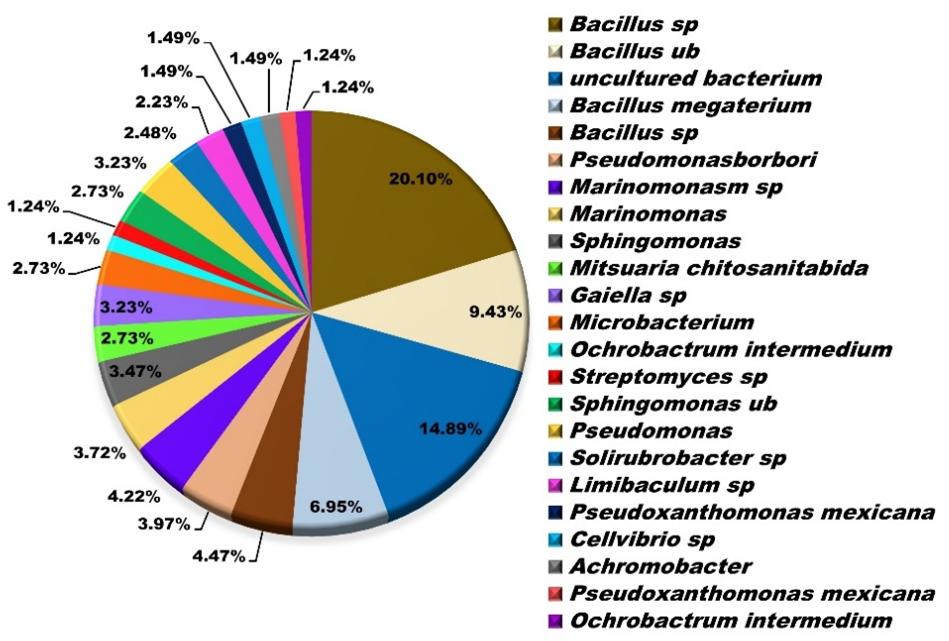

Supplement: Supplementary file 12 — High resolution image (TIF 589 kb) [file 248_2024_2435_MOESM6_ESM.tif]

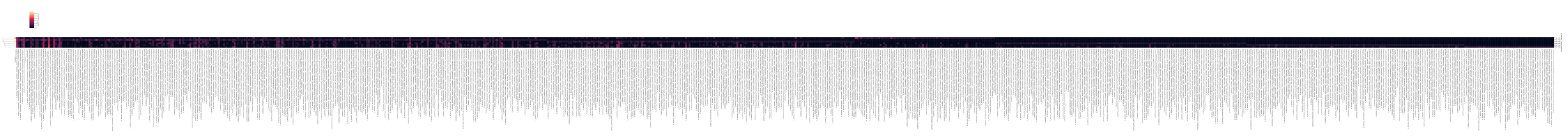

Supplement: Supplementary file 13 — Cluster heatmap for distribution of bacterial species in different treatments (Mg1 - B. velezensis VB7 + Root Knot Nematode, Mg2- B. velezensis VB7 alone, Mg3- T. koningiopsis TK + Root Knot Nematode, Mg4 - T. koningiopsis TK alone, Mg5 - B. velezensis VB7 + T. koningiopsis TK + Root Knot Nematode, Mg6 - Root Knot Nematode (M. incognita)) (JPG 6558 kb) [file 248_2024_2435_MOESM7_ESM.jpg]
